# Overlapped tobacco shred image segmentation and area computation using an improved Mask RCNN network and COT algorithm

**DOI:** 10.3389/fpls.2023.1108560

**Published:** 2023-04-17

**Authors:** Li Wang, Kunming Jia, Yongmin Fu, Xiaoguang Xu, Lei Fan, Qiao Wang, Wenkui Zhu, Qunfeng Niu

**Affiliations:** ^1^ School of Electrical Engineering, Henan University of Technology, Zhengzhou, ;China; ^2^ Xuchang Cigarette Factory, China Tobacco Henan Industry Co, Ltd, Xuchang, ;China; ^3^ Key Laboratory of Grain Information Processing and Control (Henan University of Technology), Ministry of Education, Zhengzhou, ;China; ^4^ Zhengzhou Tobacco Research Institute of China National Tobacco Company (CNTC), Zhengzhou, ;China

**Keywords:** overlapped tobacco shred, instance segmentation, area computation, Mask RCNN, Cot

## Abstract

**Introduction:**

The classification of the four tobacco shred varieties, tobacco silk, cut stem, expanded tobacco silk, and reconstituted tobacco shred, and the subsequent determination of tobacco shred components, are the primary tasks involved in calculating the tobacco shred blending ratio. The identification accuracy and subsequent component area calculation error directly affect the composition determination and quality of the tobacco shred. However, tiny tobacco shreds have complex physical and morphological characteristics; in particular, there is substantial similarity between the expanded tobacco silk and tobacco silk varieties, and this complicates their classification. There must be a certain amount of overlap and stacking in the distribution of tobacco shreds on the actual tobacco quality inspection line. There are 24 types of overlap alone, not to mention the stacking phenomenon. Self-winding does not make it easier to distinguish such varieties from the overlapped types, posing significant difficulties for machine vision-based tobacco shred classification and component area calculation tasks.

**Methods:**

This study focuses on two significant challenges associated with identifying various types of overlapping tobacco shreds and acquiring overlapping regions to calculate overlapping areas. It develops a new segmentation model for tobacco shred images based on an improved Mask region-based convolutional neural network (RCNN). Mask RCNN is used as the segmentation network’s mainframe. Convolutional network and feature pyramid network (FPN) in the backbone are replaced with Densenet121 and U-FPN, respectively. The size and aspect ratios of anchors parameters in region proposal network (RPN) are optimized. An algorithm for the area calculation of the overlapped tobacco shred region (COT) is also proposed, which is applied to overlapped tobacco shred mask images to obtain overlapped regions and calculate the overlapped area.

**Results:**

The experimental results showed that the final segmentation accuracy and recall rates are 89.1% and 73.2%, respectively. The average area detection rate of 24 overlapped tobacco shred samples increases from 81.2% to 90%, achieving high segmentation accuracy and overlapped area calculation accuracy.

**Discussion:**

This study provides a new implementation method for the type identification and component area calculation of overlapped tobacco shreds and a new approach for other similar overlapped image segmentation tasks.

## 1 Introduction

The implementation guidelines set out in Articles 9 and 10 of the WHO Framework Convention on Tobacco Control (FCTC) require the manufacturers and importers of tobacco products to disclose the contents of tobacco products to government authorities, including the type of tobacco shred and each type blending ratio of tobacco shred. Tobacco manufacturers must also have equipment and methods for detecting and measuring tobacco shred components (Acuña, 2017; Niu et al., 2022). The relative proportions of each tobacco shred type (tobacco silk, cut stem, expanded tobacco silk, and reconstituted tobacco shred) impacts the smoke characteristics, physical indicators, and sensory quality of cigarettes (State Tobacco Monopoly Administration, 2009; Chen et al., 2015). Therefore, high-precision and high-efficiency tobacco shred type identification and component determination are crucial to ensuring the quality of the tobacco shred blending process, homogeneity of production, examination of formula design, and accurate identification of tobacco products.

The detection of tobacco shred components has been extensively investigated using both manual and instrumental detection methods. The manual sorting approach involves identifying the tobacco silk, cut stem, expanded tobacco silk, and reconstituted tobacco shred varieties by means of human observation, and then calculating the proportion of various tobacco shreds after weighing. However, this technique has low detection efficiency and is to some extent subjective (Wei et al., 2022). Instrumental detection methods include tobacco shred red, green, and blue (RGB) analysis, hyperspectral imaging analysis, near-infrared spectroscopy, thermal analysis technology, cigarette smoke, anhydrous ethyl ketone, machine vision, and others. Kou et al. (2021) measured the RGB mean value of tobacco powder made from different proportions of the tobacco silk and cut stem varieties. They developed a polynomial regression model that combined the blending ratio and the RGB mean value and proposed a ratio determination method based on RGB image processing to predict cut stem components of tobacco shred. Mei et al. (2021) distinguished the sample components using the spectral data of all sample pixels and proposed a method for identifying tobacco shred components relying on hyperspectral imaging technology. Li et al. (2019) collected the near-infrared spectral data of tobacco shred samples with various component ratios, established an infrared spectral model with the partial least squares regression (PLS) method, and proposed an approach for predicting the blending uniformity of tobacco shreds using infrared spectroscopy. Zhang et al. (2019) utilized the thermogravimetric analysis method to determine the similarity of the tobacco shreds’ thermogravimetric reaction rate curves and established a method for calculating the blending uniformity of tobacco shred according to the coefficient of variation between the similarities. Ye et al. (2013) analyzed and compared the smoke indicators and conventional chemical components of tobacco shreds with varying mixing ratios to determine the differences in the blending components. Lin et al. (2020) made use of the fact that the floating rate of expanded tobacco silk under anhydrous ethyl ketone is significantly higher than that of other tobacco shred types, and subsequently developed a method for determining the proportion of components of expanded tobacco silk. Dong et al. (2015); Dong et al. (2016a); Dong et al. (2016b) acquired tobacco shred images through machine vision technology, creating a feature database using different types of tobacco shred images using RGB, HSV (hue, saturation, value) color space pixel variance, contrast, entropy, and others to determine the tobacco shred type. However, all of these detection techniques have some limitations, such as issues with destructive testing, long testing periods, and the incomplete detection of tobacco shred types.

In recent years, machine vision-based deep learning methods have provided advanced and efficient image processing solutions in agriculture. Deep learning methods, combined with machine vision technology, have been widely used in plant disease and pest classification, including the classification of fresh tobacco leaves of various maturity levels (Chen et al., 2021); the classification of tobacco plant diseases (Lin et al., 2022); the classification of wheat spike blast (Fernández-Campos et al., 2021); the classification of rice pests and diseases (Yang et al., 2021); the detection of plant parts such as tobacco leaves and stems (Li et al., 2021); the detection of tomato diseases (Liu et al., 2022); the detection of wheat head diseases (Gong et al., 2020); the detection of brown planthoppers in rice (He et al., 2020); plant image segmentation, such as tobacco planting areas segmentation (Huang et al., 2021); field-grown wheat spikes segmentation (Tan et al., 2020); rice ear segmentation (Bai-yi et al., 2020; Shao et al., 2021); rice lodging segmentation (Su et al., 2022); photosynthetic and non-photosynthetic vegetation segmentation (He et al., 2022); weed and crop segmentation (Hashemi-Beni et al., 2022); and wheat spike segmentation (Wen et al., 2022). Deep learning methods combined with machine vision technology have been utilized in research focused on the classification of tobacco shred images. Gao et al. (2017) proposed a method for identifying tobacco shreds using convolutional neural networks that is based on differences in the structural characteristics of various tobacco shreds. Zhong et al. (2021) built a recognition model utilizing a residual neural network and optimized the model’s pre-training weights, optimization algorithms, and learning rates. They found that both the accuracy and recall rate of the trained model were higher than 96%. Niu et al. (2022) used ResNet50 as the network’s primary framework and optimized it by increasing the multi-scale structure, in turn optimizing the number of blocks and loss function. Their experimental results showed that the network’s tobacco shred classification accuracy was 96.56%.

In the above research on tobacco shred image classification methods, tobacco shred classification was always carried out using single tobacco shred image samples. In practice, tobacco blends found on the quality inspection line will inevitably contain different types of overlapping and stacked tobacco shreds. The object detection and segmentation methods of overlapping and stacked tobacco shred images have rarely been investigated. However, the type identification and component determination of overlapped and stacked tobacco shred directly affect the calculation accuracy of the blending ratio of tobacco shred components, which is a crucial aspect of research.

The object detection and segmentation methods of overlapped images using machine vision technology have been studied in some fields. Fan et al. (2020) developed a 3D-Mask region-based convolutional neural network (3D-Mask RCNN) for the mass detection and segmentation of overlapping tissue during screening. The 3D-Mask RCNN achieved an average precision (AP) of 0.934 and a false negative rate (FNR) of 0.053. Wang and He (2022) focused on the overlapped images of covered apples in an orchard to perform accurate segmentation. Taking 3D-Mask RCNN as the segmentation network’s mainframe, the attention mechanism was added to enhance the network’s ability to extract features. The model achieved a recall rate, precision rate, F1 score, and segmentation mean average precision (mAP) of 97.1%, 95.8%, 96.4%, and 0.917, respectively. Yu et al. (2019) achieved accurate segmentation and picking point positioning for overlapping strawberries with a 3D-Mask RCNN and localization algorithm. The average detection precision rate was 95.78%, the recall rate was 95.41%, and the mean intersection over union (MIoU) rate, for instance segmentation, was 89.85%. Qi et al. (2022) concentrated on detecting dense occlusion and overlapped images of auxiliary equipment in an engine room using SsdNet as a mainframe network while adding repulsion loss. The mAP reached 78.95%, which was 5.63% higher than the original SsdNet (SSD). Wen D et al. (2022)’s study set out to identify overlapping bubbles in high void fraction conditions with the use of a convolutional neural network (CNN), and their algorithm reached 85% accuracy under high overlap rate conditions. Wu et al. (2022) employed a residual U-Net network to detect overlapped immunohistochemistry-positive cells in the proposed dataset. Their technique detected 86.04% of the overlapped cells, and the proposed genetic algorithm (GA) outperformed the baseline methods. Su et al. (2019) focused on overlapped ship detection in high-resolution synthetic aperture radar (SAR) imagery using a modified version of the RetinaNet network. The final *AP* ^50^ reached 94.2%. Prasetyo et al. (2020) investigated the performance of two CNN-based segmentation methods, that is, YOLO (you only look once) and Mask RCNN (mask region-based conventional neural network), for separating the heads and tails in images of fish with high variability in terms of their background and illumination, and with overlapping objects. YOLO was high performing, as shown by its 98.6% and 96.73% precision rates. Jia et al. (2020) proposed a model for harvesting robot vision detectors utilizing Mask RCNN to realize the recognition and segmentation of overlapped apples. The precision and recall rates were 97.31% and 95.70%, respectively. Zhang et al. (2022) developed a mask-labeling methodology for particles with a varying degree of overlap that can establish a large and diverse training set without manual labeling. This could be an efficient sample-labeling method.

Regarding the image segmentation of overlapped tobacco shreds, the small size of single-tobacco shreds, their various shapes, and tiny tobacco shreds have complex physical and morphological characteristics, with little difference in macro-scale features between the tobacco silk and expanded tobacco silk varieties, and this complicates the identification and classification of single tobacco shreds with machine vision technology. Furthermore, there are 24 overlapped tobacco shreds derived from four distinct types. The self-winding varieties are more difficult to separate than overlapped types, posing considerable challenges for the segmentation tasks of images of overlapped tobacco shreds and the subsequent calculation of the component area.

This study proposes an overall solution based on an improved Mask RCNN instance segmentation model and an algorithm for the area calculation of overlapped tobacco shred region to identify overlapped tobacco shred types and calculate the area of the overlapped region. The focus is on the identification of overlapped tobacco shred types, as our research object is determining the best method of identifying tobacco shred components for real-world use in field quality inspection lines. This study’s contributions to this research area are as follows:

- Establishing two types of original overlapped tobacco shred image datasets, 920 common objects in context (COCO) and 920 visual object classes (VOC). The two datasets consist of images captured from four tobacco shred varieties with 24 overlapped types. The raw overlapped tobacco shred datasets are initially established and applied in a tobacco field, avoiding overfitting and field-specificity.
- Developing an accurate Mask RCNN model to achieve overlapped tobacco shred detection and segmentation utilizing digital images. Segmentation models were developed and compared using the SsdNet, Deeplap_v3, FcnNet, and RetinaNet architectures with the chosen datasets. The constructed improved Mask RCNN network (Densenet121, U-FPN, anchors parameters) demonstrated the highest instance of segmentation accuracy. It provides good segmentation capability for overlapped tobacco shred images with different sizes and types, outperforming other similar segmentation models.
- Proposing a calculation of overlapped tobacco shred region (COT) algorithm to be first applied to overlapped region identification and overlapped area calculation. This algorithm accurately detects and calculates areas in the images of overlapping tobacco shred, and effectively avoids the negative optimization situation of identifying and calculating overlapped areas.
- Providing a new implementation method for the identification of tobacco shred type and component area calculation of overlapped tobacco shreds and a new approach for other similar overlapped image segmentation tasks.

## 2 Data acquisition and preprocessing

### 2.1 Data collection

**Figure 1:**
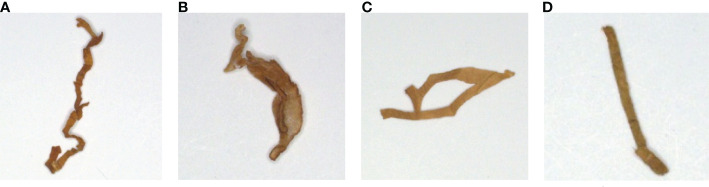
The four tobacco shred varieties: **(A)** tobacco silk; **(B)** cut stem; **(C)** expanded tobacco silk; and **(D)** reconstituted tobacco shred.

Cigarette samples in this study were obtained from the Xuchang Tobacco Research Institute of the China National Tobacco Corporation. Each cigarette was a mixture with a certain ratio of four tobacco shred varieties, that is the tobacco silk, cut stem, expanded tobacco silk, and reconstituted tobacco shred varieties (shown in **Figure 1**). Cigarettes were randomly selected from a specific brand, and thereafter all blended tobacco shreds were obtained. Tobacco shred from a batch with a known serial number was inserted into the vibration device, and then vibration experiments were performed.

**Figure 2:**
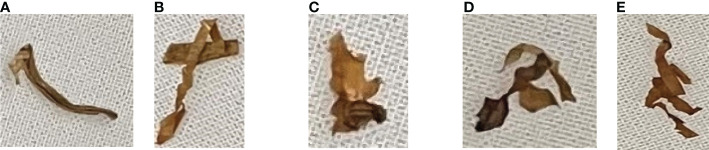
The vibrated tobacco shred types: **(A)** single tobacco shred; **(B)** self-winding tobacco shred; **(C)** adhesion tobacco shred; **(D)** inter-overlapped tobacco shred; and **(E)** stacked tobacco shred.

The classification results of the vibrated tobacco shred through the vibration experiment are shown in **Figure 2**. In this experiment, tobacco shred was categorized as (A) single tobacco shred; (B) self-winding tobacco shred; (C) adhesion tobacco shred; (D) inter-overlapped tobacco shred; or (E) stacked tobacco shred. Hence, the overlapped tobacco shreds were defined as one of three overlapped types, namely (A) self-winding tobacco shreds (based on their length); (B) adhesion tobacco shreds (the borders of the two tobacco shreds were connected); or (C) inter-overlapped tobacco shreds, (there was tendency for overlap between two tobacco shreds).

**Figure 3:**
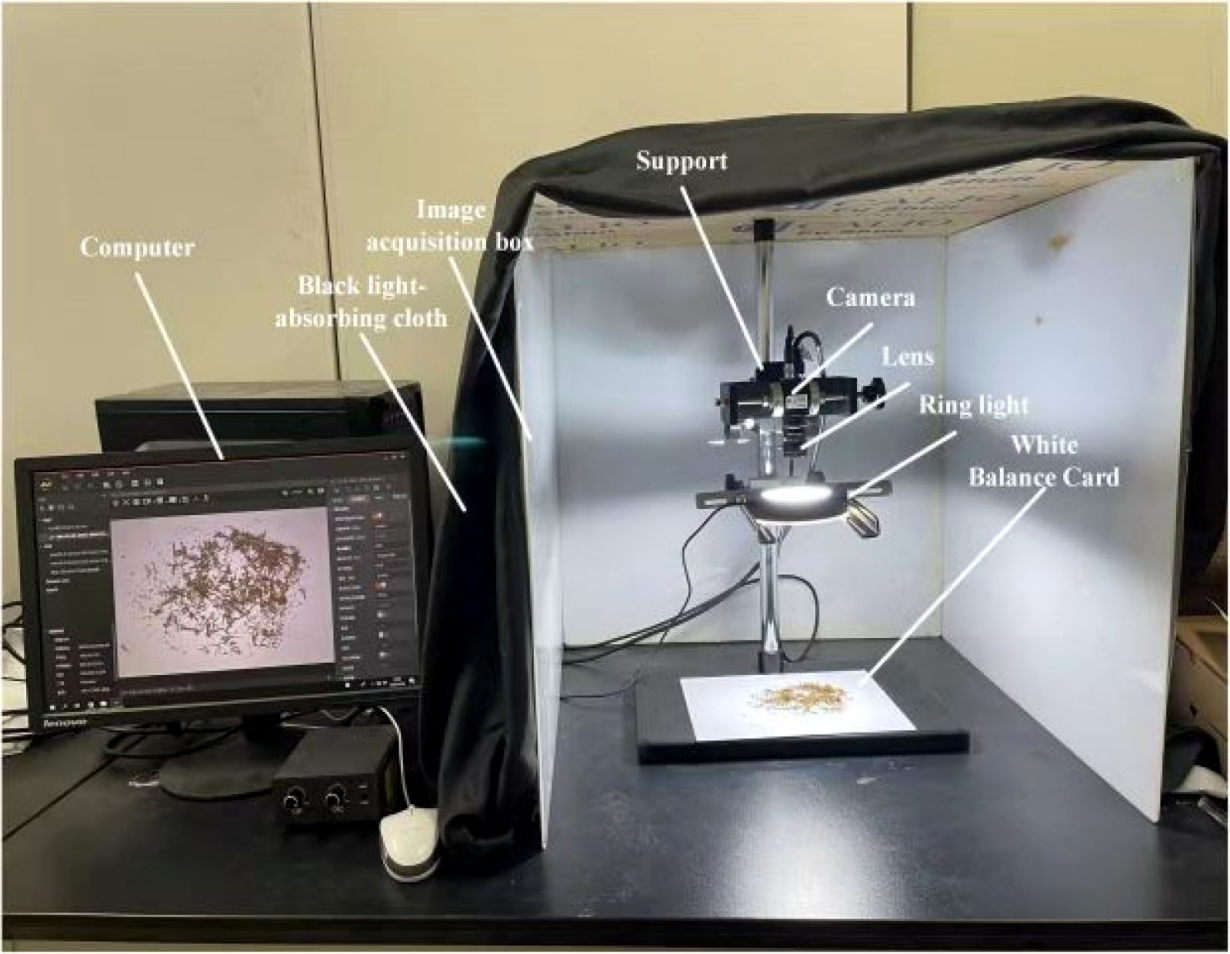
The image acquisition system.

An image acquisition darkroom with four photographic reflectors was designed to obtain high-quality tobacco shred images. **Figure 3** is a photograph of an image acquisition system (Niu et al., 2022). The camera and light source were fixed on the bracket, including the universal light source lighting frame, the settings of which could be changed with a fine-tuning knob attached to a 600 mm threaded rod. A Hikvision MV-CE100-30GC industrial camera, a 10-megapixel color camera, was used and equipped with a MVL-HF1224M-10MP 12 mm focal length Hikvision industrial lens. The Hikvision Technology Industrial Ring Angle Light Source R120-80-25 was selected as a ring light source to provide uniform brightness in the shooting field of view, eliminating the influence of shadows cast by the tobacco shreds. The computer and the industrial camera were connected by a network cable, ensuring that the tobacco shred images were transmitted over a stable connection at high speed.

**Table 1** shows the overlapped tobacco shred types and the number of images utilized in this study. The overlapped tobacco shred images were obtained from the four tobacco shred varieties [cut stem (G), expanded tobacco silk (P), tobacco silk (Y), and reconstituted tobacco shred (Z)]. A total of 920 overlapped tobacco shred images of 24 overlapped tobacco types (i.e., four self-winding tobacco shreds, 10 adhesion tobacco shreds, and 10 inter-overlapped tobacco shreds) were taken. The size of a single image was 3,840 × 2,748 pixels. The number of tobacco shred images taken differs for various overlapped tobacco shreds. For the tobacco shred overlaps of the same type (for example, GG is overlapped with cut stem and cut stem), the ratio of self-winding, to adhesion, to inter-overlapped types was set to 1:1:2. For the overlap of different types of tobacco shred (for example, GP is cut stem overlapped with expanded tobacco silk), the ratio of adhesion to inter-overlapped types was set to 1:1. As sample images could not easily be obtained, and a wide variety of overlapped types exist, the ratio of the training set to the testing set was set to 8:2.

**Table 1:** Overlapped tobacco shred datasets.

Overlapped tobacco shred types	Self-winding tobacco shred	Adhesion tobacco shred	Inter-overlapped tobacco shred	Number of images
GG	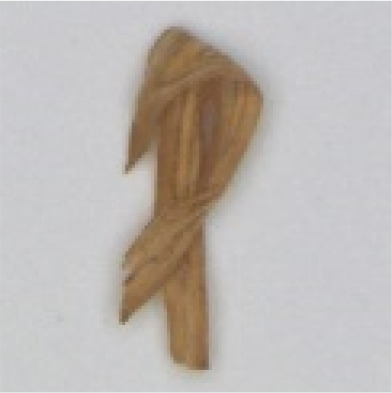	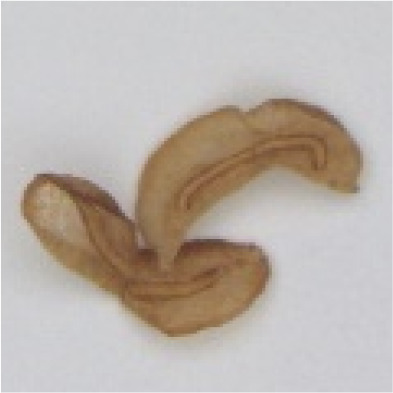	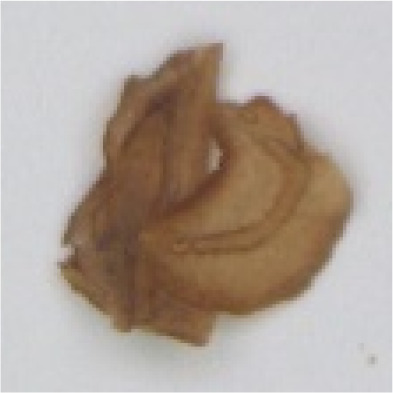	98
GP		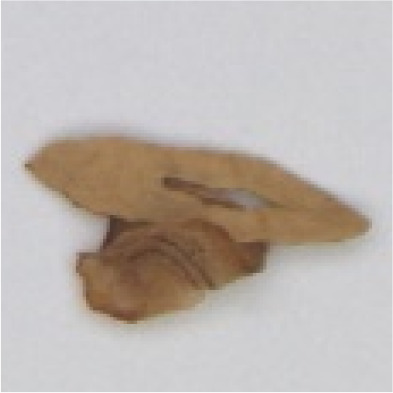	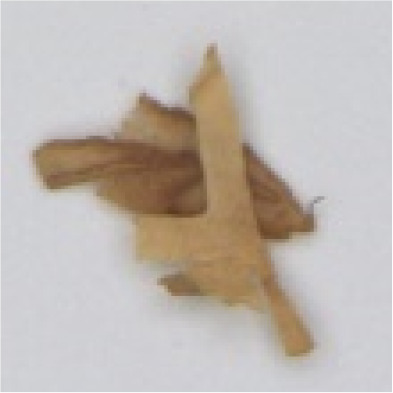	96
GY		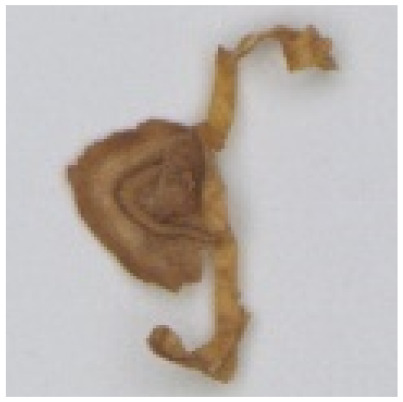	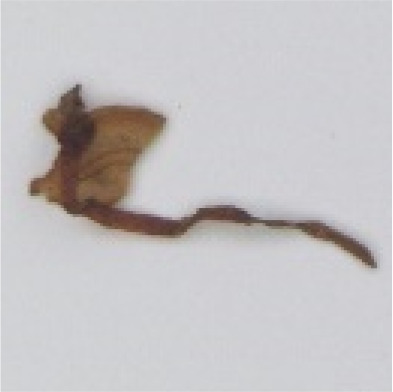	80
GZ		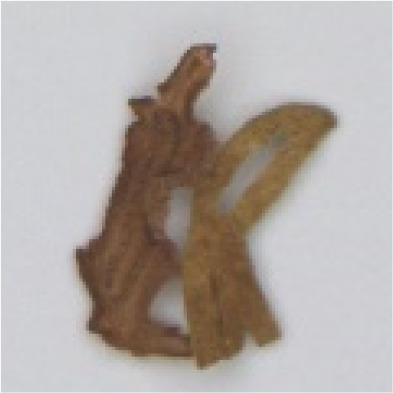	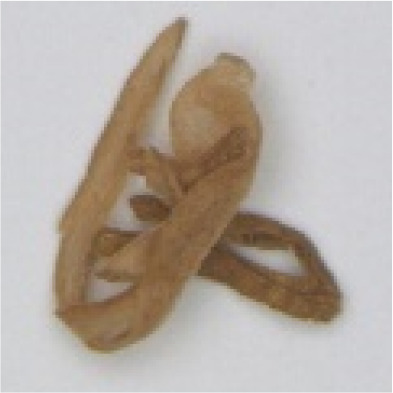	95
PP	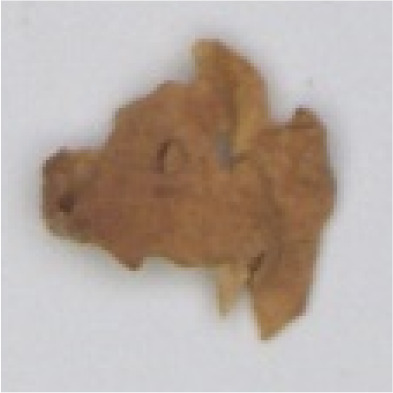	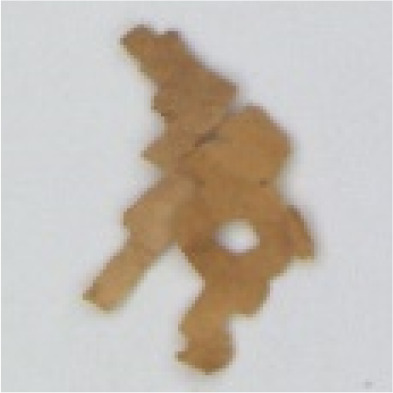	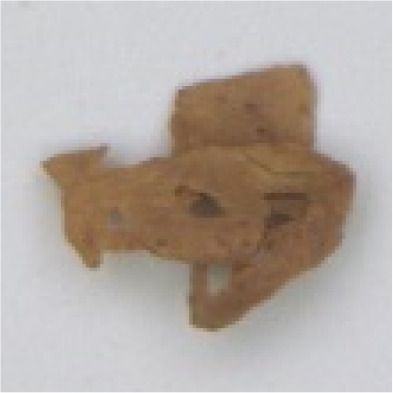	95
PY		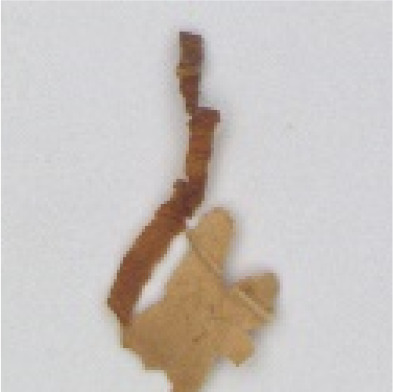	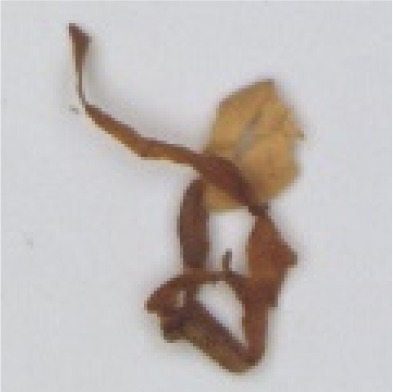	89
PZ		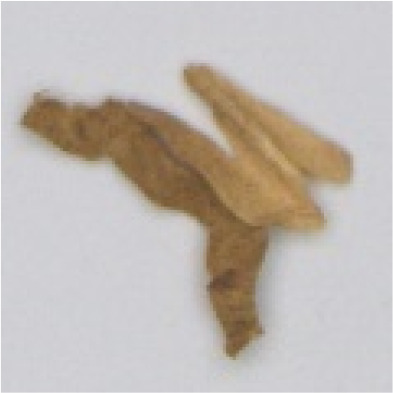	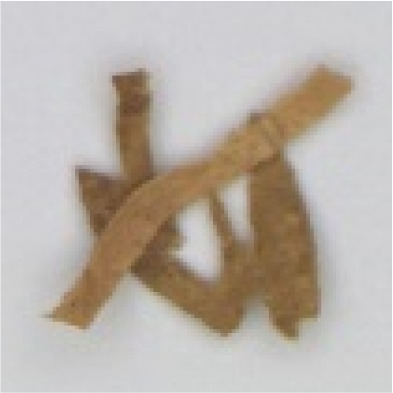	97
YY	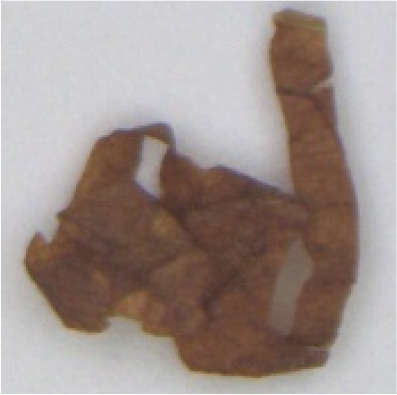	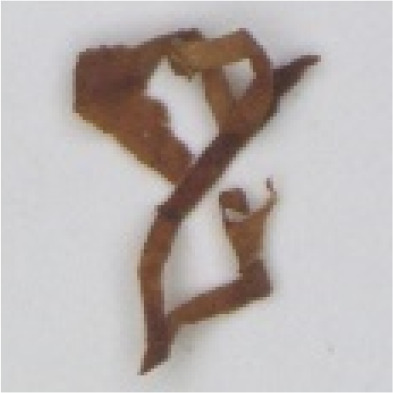	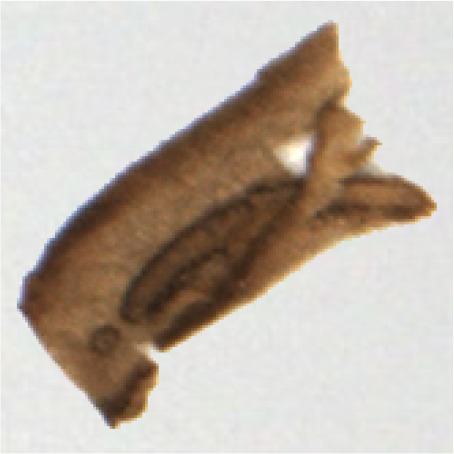	91
YZ		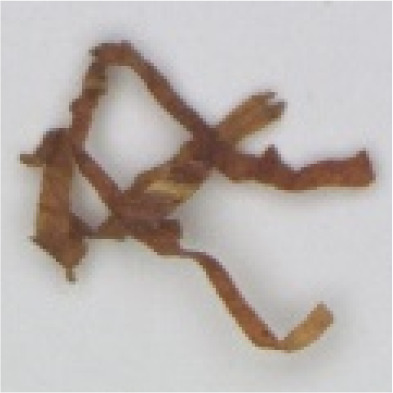	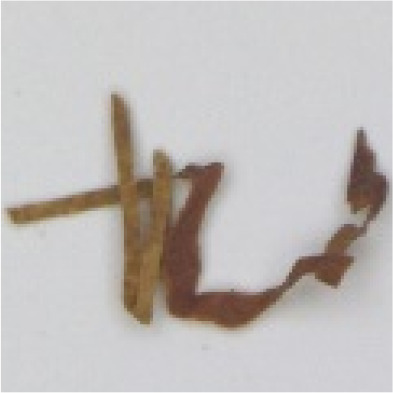	93
ZZ	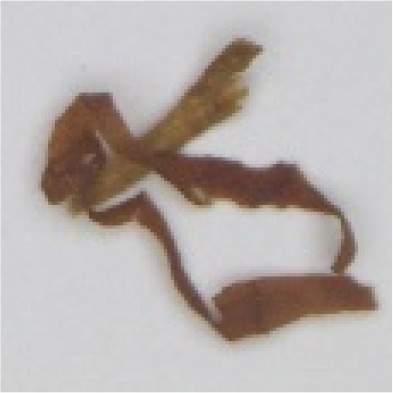	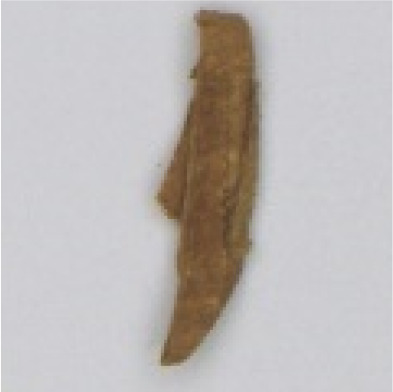	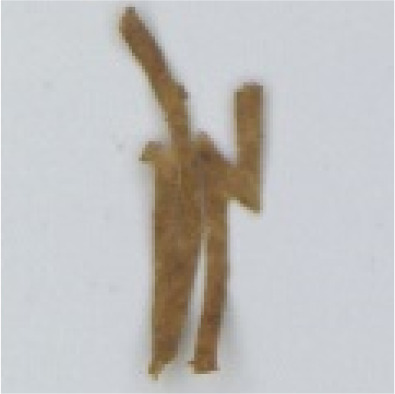	86
Total				920

GG, cut stem and cut stem; GP, cut stem and expanded tobacco silk; GY, cut stem and tobacco silk; GZ, cut stem and reconstituted tobacco shred; PP, expanded tobacco silk and expanded tobacco silk; PY, expanded tobacco silk and tobacco silk; PZ, expanded tobacco silk and reconstituted tobacco shred; YY, tobacco silk and tobacco silk; YZ, tobacco silk and reconstituted tobacco shred; ZZ, reconstituted tobacco shred and reconstituted tobacco shred.

### 2.2 Data preprocessing

**Figure 4:**
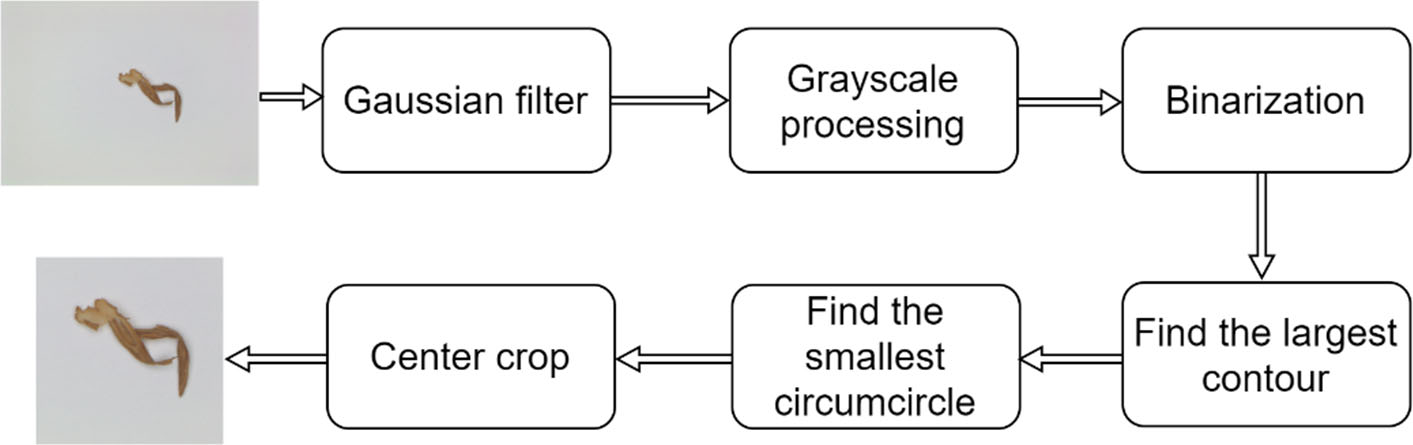
Overlapped tobacco shred image preprocessing process.

It was observed that the segmentation model’s training and testing times increased considerably if the tobacco shred object in the overlapped tobacco shred images obtained by the image acquisition system was small, and if the images contained a lot of background information. The image preprocessing approach starts by treating the overlapped tobacco shred images with the OpenCV algorithm, then finding the minimum circumscribed circle of the object and cutting the contour. The algorithm flow chart is shown in **Figure 4**. The image preprocessing algorithm ensures that the invalid background information in the picture is reduced by preserving the foreground information of the image, thereby significantly reducing the size of the image.

**Figure 5:**
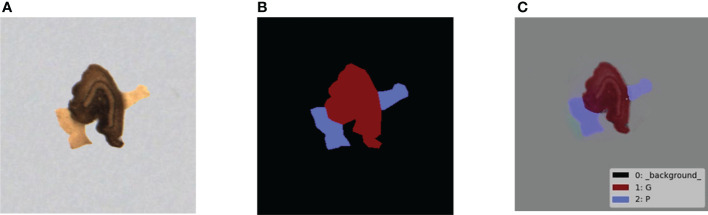
Overlapped tobacco shred example of instance segmentation: **(A)** original image; **(B)** mask image of instance segmentation; and **(C)** visualization of the mask image. G, cut stem; P, expanded tobacco silk.

The preprocessed overlapped tobacco shred images were labeled using LabelMe, an image annotation tool, to generate corresponding mask images. Thereafter, the COCO official datasets were used to develop the code and the overlapped tobacco shred datasets of the COCO data type. The four tobacco shred regions in the image were marked, and the rest were taken as the image’s background. The labeled image of the inter-overlapped GP is shown in **Figure 5**.

## 3 Methods

### 3.1 Segmentation method

There are three main challenges in the task of overlapped tobacco segmentation:

(1) The size of a single tobacco shred is small, the shape of the tobacco shred is variable, and tiny tobacco shreds have complex physical and morphological characteristics; in particular, there is substantial similarity between expanded tobacco silk and tobacco silk.
(2) There are 24 types of overlapped tobacco shreds that can be extracted from the four different varieties, and shreds of the self-winding type are not easily distinguished.
(3) Overlapped tobacco shreds are small target objects, meaning that their segmentation is difficult.

The following approaches were used to overcome the above challenges:

- Mask RCNN is a state-of-the-art CNN-based method in which the detection and segmentation of objects are performed simultaneously to address broad problems associated with overlap across multiple domains. Therefore, Mask RCNN, as the mainframe of the segmentation network, was used to complete the target detection of the overlapped tobacco shreds in the image and segment various types of tobacco shreds.
- In the case of complex classification tasks and limited datasets, the Resnet in the backbone of the Mask RCNN was replaced with Densenet121, because the latter adopts a dense connection mode between layers. As a result, multiple rounds of shallow information were used to increase the ability of the Mask RCNN to extract tiny features in the shallow information of the overlapped tobacco shreds. The Densenet121 can achieve better performance using fewer datasets compared with the Resent.
- Because of the rich shallow features in the tiny overlapped tobacco objects, the deep features contain less target information. Accordingly, the feature pyramid network (FPN) in Mask RCNN was changed to a U-FPN. In contrast to the FPN, U-FPN performs feature multiplexing on C2 and C3 and features multiplexing on P2, P3, P4, and P5, which significantly enhances the utilization rate of tiny features in shallow information.
- The anchors parameters in the region proposal network (RPN) were optimized, and the size and aspect ratios suitable for the small objects of overlapped tobacco shred were designed to ensure that the RPN could extract region of interest (ROI) features from different levels efficiently. In this way, the extraction and bounding boxes performance of small objects was significantly improved without a correspondingly large increase in the computational cost, and this increased the model’s ability to detect tobacco shred and enhanced its accuracy.

### 3.2 Segmentation network

#### 3.2.1 Overall model framework of improved Mask RCNN

The Mask RCNN network was introduced by He et al. (2017). The network has achieved excellent results in various tasks by utilizing the Microsoft Common Objects in Context (MS COCO) dataset, including object detection, instance segmentation, and keypoint detection. The mainframe of the segmentation network in this paper adopts the improved Mask RCNN network. The network structure of the improved Mask RCNN network is composed of main modules with backbone (CNNs and FPNs), RPNs, ROI aligns, fully convolutional networks (FCNs), and fully connected (FC) layers.

**Figure 6:**
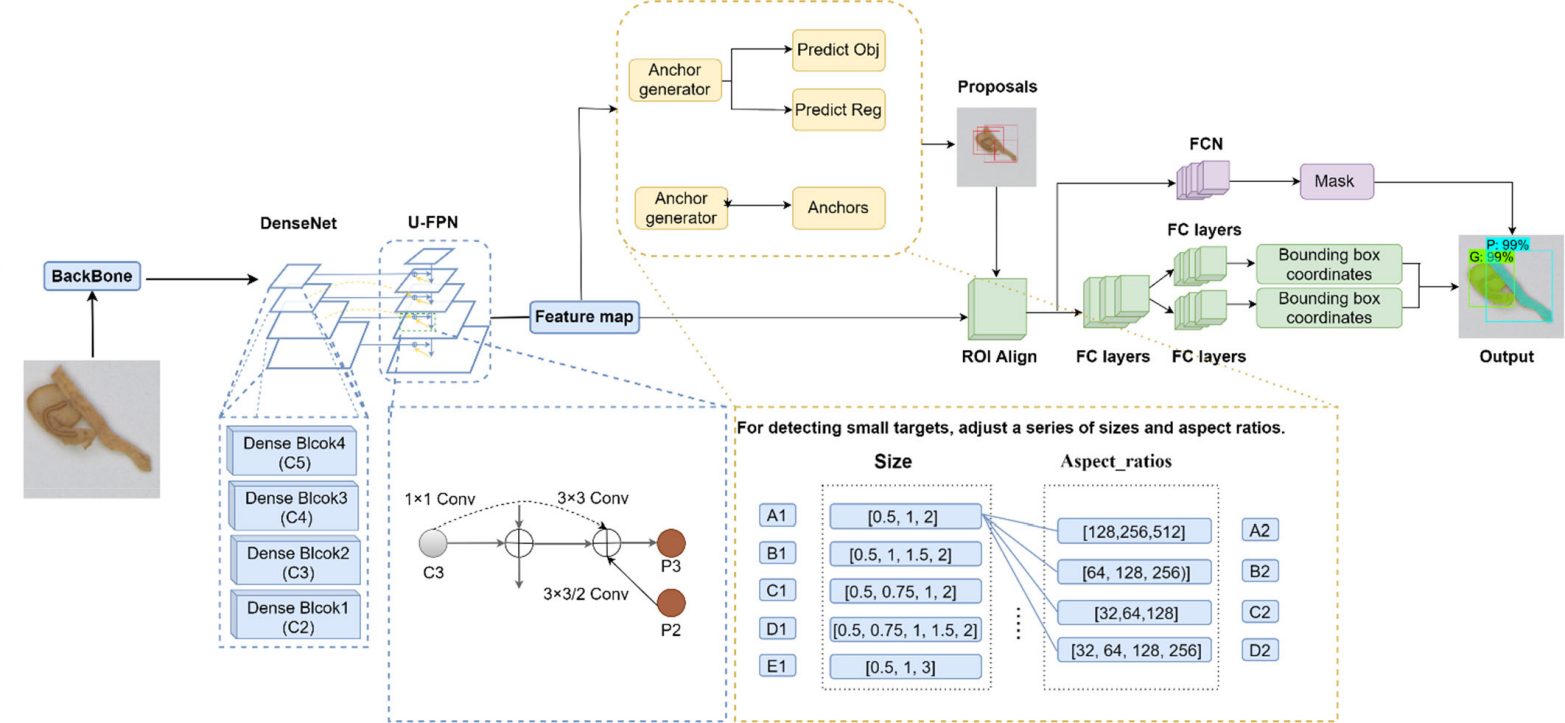
The overall framework of the improved Mask RCNN. FC layers, fully connected layers; FCNs, fully convolutional networks; Mask RCNN, mask region-based conventional neural network; ROI align, region of interest align.

The overall framework of the improved Mask RCNN network is shown in **Figure 6**. It can be seen that the model input size is 500 × 500 pixels of overlapped tobacco shred images, and that the backbone network uses the DenseNet121+U-FPN group to perform feature extraction that obtains the feature map. Moreover, the feature map output from the backbone is fed to the RPN to generate proposals. Subsequently, the ROI output from the RPN is mapped to extract the corresponding overlapped tobacco shred features in the shared feature map. Finally, the instance segmentation of overlapped tobacco shred images is completed with FC layers and FCNs. The model’s output is the overlapped tobacco shred type.

#### 3.2.2 Improved Mask RCNN performance brought about by a change in backbone

**Figure 7:**
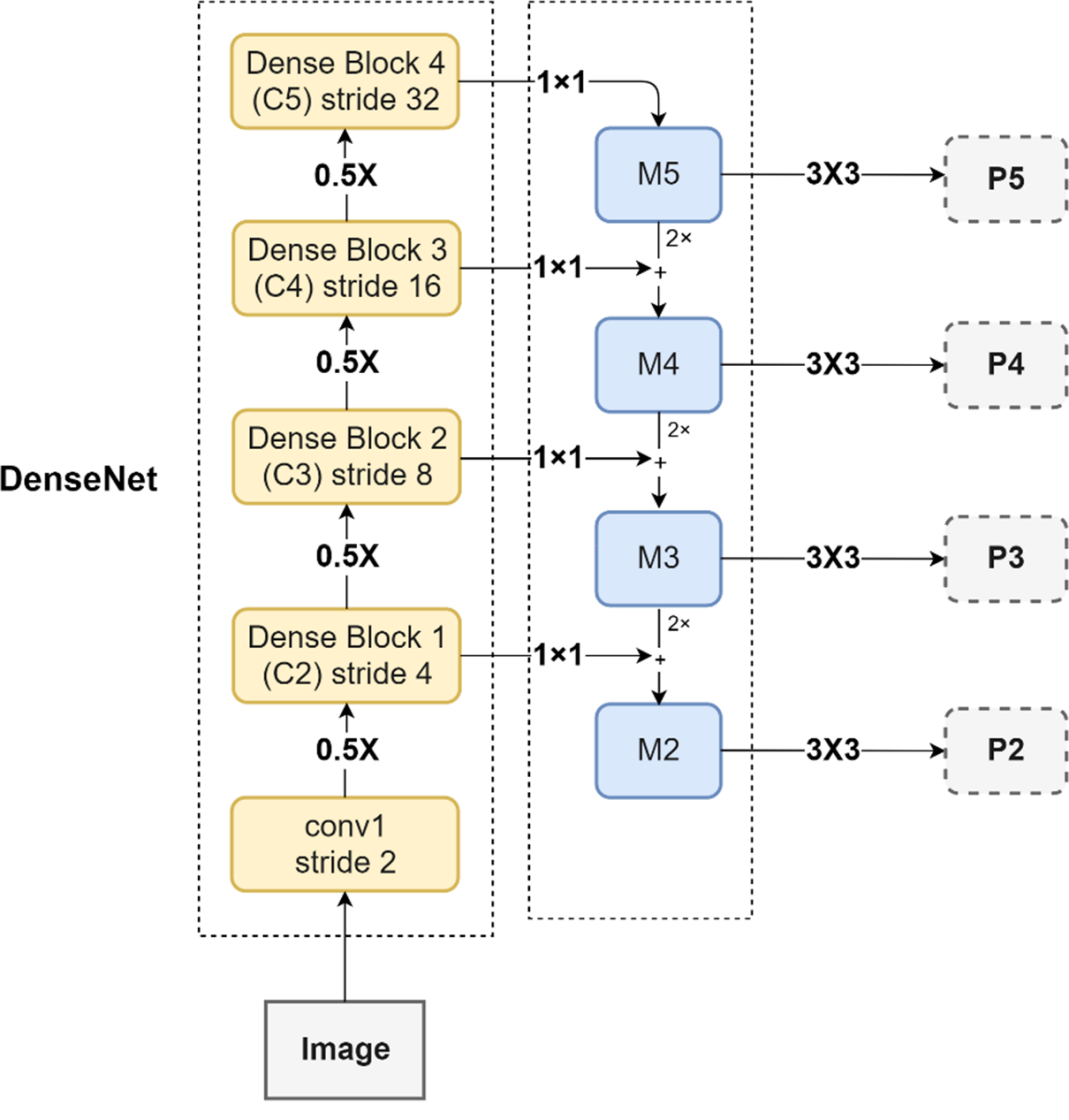
A new type of backbone (Densenet121+FPN).

Because of the different morphological characteristics and the slight differences in the features of the overlapped tobacco shreds, extracting the features of different overlapped tobacco shreds is challenging, particularly in the overlapped region. In the Mask RCNN network, using Resnet50 to extract different levels of features from the overlapped tobacco shred input images is less effective. However, an increased number of Densenet121 network layers can enhance the ability to extract small target detail information of different tobacco shreds. The dense connection form can effectively extract the small differences in the features, and features of different tobacco shreds in the overlapped regions. Therefore, DenseNet121 can effectively extract small-sized target detail information and small features in larger-sized overlapped tobacco shreds, which enhances the overall feature extraction ability of the model in regions with overlapped tobacco shred, and to a certain extent solves the problem of shallow feature loss. Densenet121 was set to four feature extraction layers, that is Dense Block 1, Dense Block 2, Dense Block 3, and Dense Block 4. The four feature return values have undergone different downsampling times (i.e., 2, 3, 4 and 5s) and lateral connections. Accordingly, Densenet121 constitutes a new type of backbone, as shown in **Figure 7**.

#### 3.2.3 Improved Mask RCNN about U-FPN

**Figure 8:**
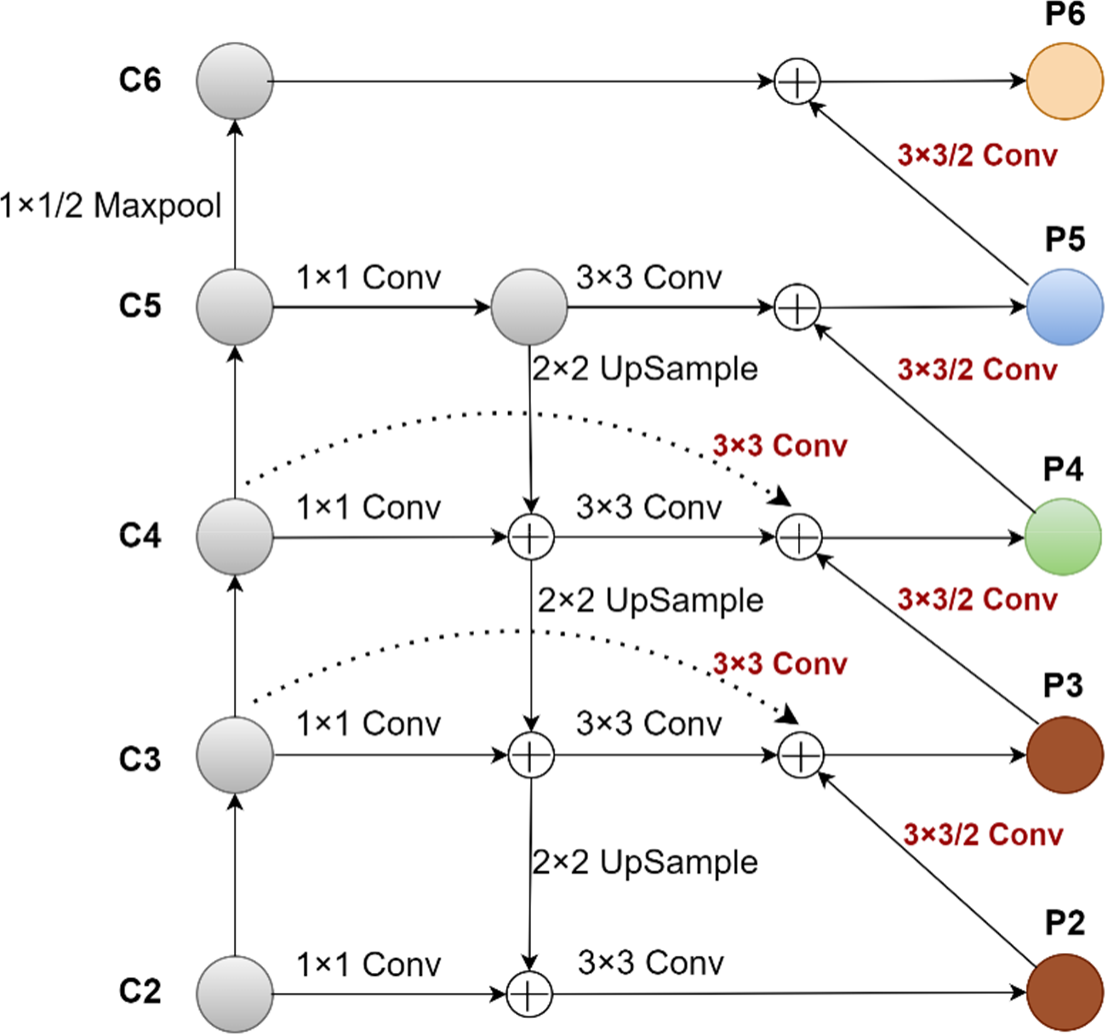
The U-FPN network structure.

After being extracted by the CNN network, the receptive field of the shallow-feature map is small, and more detailed information about the tobacco shred targets is produced, whereas the receptive field of the deep-feature map is large, and the information it produces about these tiny targets is less detailed. Although the top-down and same-layer connected structure of the FPN combines deep and shallow features to a certain extent to meet the needs of subsequent classification and detection of overlapped tobacco shreds, it still cannot make up for the complete usage of small features in the shallow parts. The shallow feature information of small targets overlapped tobacco shred detection is rich and vital. Therefore, a bottom-up and horizontal feature multiplexing structure was added based on the top-down structure, and the shallow information was transmitted to each feature layer (P3, P4, P5, and P6), which enhanced the effective use of shallow feature information. The modified FPN network structure was named U-FPN, as shown in **Figure 8**. P3 in **Figure 8** indicates that adding P2 and C3 to P3 through 3 × 3/2 Conv and 3 × 3Conv helped obtain the shallow information of the P2 layer, and that incorporating the C3 layer into the P3 layer enhanced the fusion of the general shallow feature information.

#### 3.2.4 Improved Mask RCNN about RPN

In the Mask RCNN model, the scales and aspect ratios of the anchor were set to [128, 256, 512] and [1:1, 1:2, 2:1], respectively, with nine reference anchors being set for each position on the feature map. The RPN selects and adjusts anchor output ROIs according to the features of each stage. For instance, in P2, the layer’s feature map was 256 × 256, and the step was 4; hence each pixel on P2 generated a 4 × 4 anchor frame with an area of 16 based on the current coordinates. According to the scales and aspect ratios of the anchor, bounding boxes of the three sizes and three shapes were generated at each pixel point. The foreground and background classification and offset regression of the bounding boxes were conducted after two convolution layers.

The minor anchor scale in the Mask RCNN was 128 × 128, but there were many small targets in the overlapped tobacco shred, some of which were much smaller than this scale, resulting in the model’s inability to detect this target object. Ideally, the smaller the target, the denser the anchors to cover all the candidate regions, and the larger the target, the fewer and sparser the anchors should be. Otherwise, the anchors overlap and cause redundant computation. However, the anchors parameter set in Mask RCNN, focusing on small targets in the images of overlapped tobacco shred objects, led, to a certain extent, to the anchors for small targets being few and sparse, and the anchors for detecting large targets being many and dense. In this case, the detection performance of small objects can be significantly improved by adjusting appropriate anchor scales and aspect ratios without greatly increasing the amount of computation.

According to the statistics of the aspect ratios in each batch of images and the pixel size for tobacco shred of [0, 0.5, 0.6, 0.8, 1.0, 1.3, 1.5, 2.0], a series of anchor scales and aspect ratios were designed as [128, 256, 512], [64, 128, 256], [32, 64, 128], [32, 64, 256] and [0.5, 1, 2], [0.5, 1, 1.5, 2], [0.5, 0.75, 1, 2], [0.5, 0.75, 1, 1.5, 2], and [0.5, 1, 3]. Finally, the anchor scale and size were experimentally determined as [32, 64, 128, 256] and [0.5, 1, 1.5, 2], respectively.

### 3.3 Area calculation of overlapped tobacco shred algorithm

Based on the improved Mask RCNN network, the above detection model can effectively achieve instance segmentation for overlapped tobacco shreds with different shapes and forms and obtain the contours for the overlapped tobacco shred targets. Based on this, it is possible to calculate the pixel area and respective area proportions of the corresponding tobacco shreds for the mask image through the OpenCV algorithm. However, the overlapped region of the occluded tobacco shred cannot be obtained. The omission of the overlapped area in the covered tobacco shred directly leads to errors in the calculation of the respective areas for different tobacco shreds and the total area in the tobacco shred group during the subsequent determination of its components.

**Figure 9:**
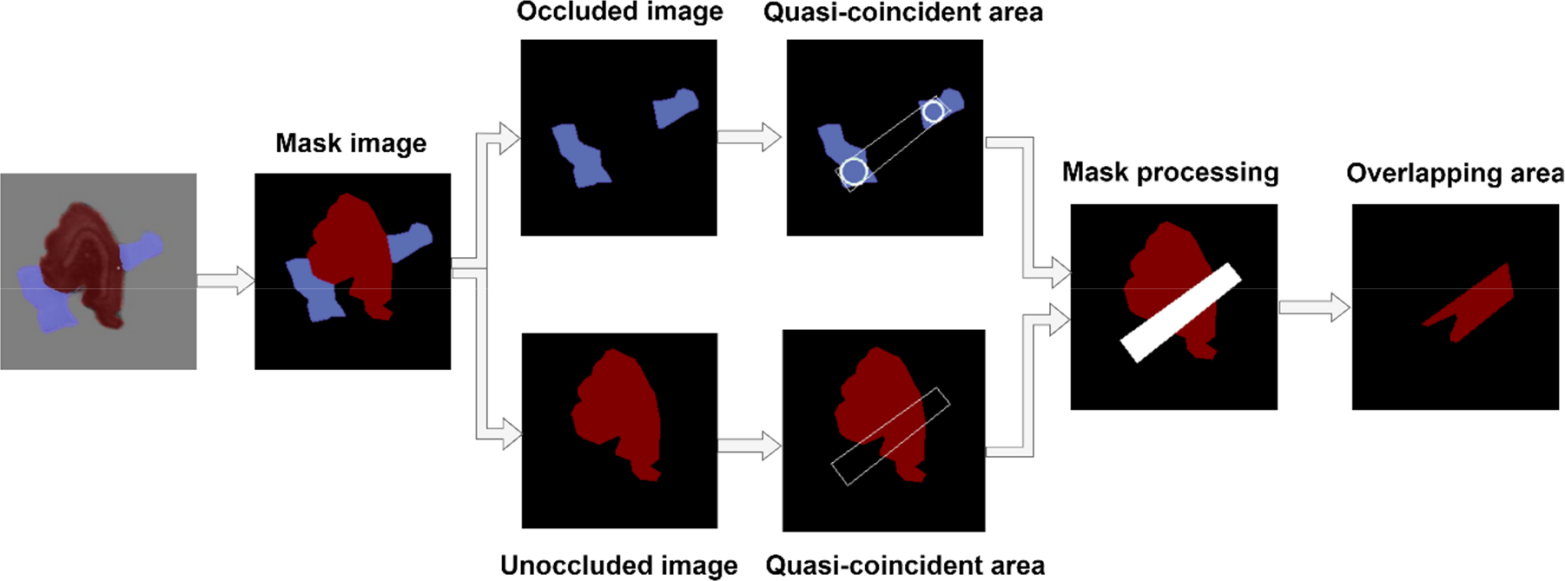
Specific process of the calculation of overlapped tobacco shred region (COT) algorithm.

The area calculation of overlapped tobacco shreds requires using the improved Mask RCNN network to generate a mask image of the overlapped tobacco shred, determine the occluded tobacco shred, draw and fit the overlapped region according to the distribution of the occluded overlapped tobacco shred, and determine the actual overlap region with the fitted overlapped region and the unoccluded area. Finally, the pixel area in the overlapped part is calculated. The algorithm for area calculation in the overlapped region, named the COT algorithm, is as follows, and the specific process is shown in **Figure 9**:

- Determine the occluded tobacco shred object. First, grayscale and binarize the mask image, and calculate and count the number of tobacco shred contours. A single contour is an unoccluded tobacco shred (see unoccluded image in **Figure 9**). Multiple contours are occluded tobacco shreds (see occluded image in **Figure 9**).
- Fit the overlapped region in the tobacco shred (see Mask processing in **Figure 9**). The multiple contours of the shrouded tobacco shred are cyclically judged, and two contours are found according to the size of the contour area. First, use the cv2.pointPolygonTest and cv2.minMaxLoc in the OpenCV function to construct the smallest rectangle inside the outline. Then draw the smallest inscribed circle round _1 with the center of the rectangle as the dot, the side length as the diameter, the center as (x1, y1), and the diameter as d1. Then, draw the smallest inscribed circle round _2 of the second contour, whose center is (x2, y2), and whose diameter is d2. Connect the centers (x1, y1) and (x2, y2) of the two inscribed circles, and draw a straight line L0. By extending the straight line L0, draw outer tangent lines L1 and L2 with the diameter of each circle for round _1 and round _2. Draw the fitted overlap region trapezoid _1 according to L1 and L2 (see the quasi-coincident area in **Figure 9**).
- Determine the overlapped region of the actual tobacco shred and pixel area by calculating the overlapped region. First, use cv2.fillPoly function to generate a mask image for the fitting area, then perform a mask operation according to the outline of the unoccluded image and find the overlapped area (see the overlapped areas in **Figure 9**). Finally, the pixel area of overlapped area is calculated using OpenCV

### 3.4 Evaluation index

#### 3.4.1 Improved Mask RCNN

The COCO evaluation index is the current mainstream target detection and instance segmentation evaluation index. This paper uses the COCO evaluation indicators, i.e., training time (T-time) and prediction time (P-time) as evaluation indicators for improved Mask RCNN and other baseline segmentation models. As an image of overlapped tobacco shreds is a small target object, among the COCO evaluation indicators, six indicators (*AP*,*AP* ^50^,*AP* ^75^,*AP^s^*,*AR* ^10^ and *AR^s^*) were selected for network performance evaluation, where *AP* ^50^ and *AR^s^* represent the precision and recall rates, respectively. The higher these values, the more ideal the segmentation model (Tong et al., 2020).

Average precision (AP):

*AP* % *AP* at IOU = 0.50: 0.0.5: 0.95 (primary challenge metric)
*AP* ^50^ % *AP* at IOU = 0.50 [PASCAL Visual Object Classes (PASCALVOC) metric]
*AP* ^75^ % *AP* at IOU = 0.75 (strict)

AP Across scales L:

*AP^s^* % *AP* for small objects: area< 32^2^

Average recall (AR):

*AR* ^10^ % *AR* give 10 at detections per image
*AR* Across scales:
*AR^s^* % *AR* for small objects: area< 32^2^

#### 3.4.2 COT algorithm

This paper uses Eqs 1–4 to calculate the actual area ratio (AAR), COT area ratio (CAR), average actual area ratio (Avg_AAR), and average COT area ratio (Avg_CAR) as evaluation indicators of the COT algorithm. The higher the CAR and Avg_CAR values, the higher the COT algorithm’s area calculation accuracy to compensate for the overlapped region of occluded tobacco shred and the more ideal the algorithm.


(1) $$AAR=\frac{AD}{Area\_1+Area\_2}$$

(2) $$CAR=\frac{AD+CD}{Area\_1+Area\_2}$$

(3) $$Avg\_AAR=\frac{\sum_{i=1}^{n}AAR_{i}}{n}$$

(4) $$Avg\_CAR=\frac{\sum_{i=1}^{n}CAR_{i}}{n}$$

AAR actual area ratio

CAR COT area ratio

AD actual detection area

CD detection area

Area_1 the pixel area of tobacco shred 1

Area_2 the pixel area of tobacco shred 2

Avg_AAR average actual area ratio

Avg_CAR average COT area ratio

*n* total number of AAR or CAR

The overlapped tobacco shred samples consist of four randomly combined tobacco shreds in pairs. Tobacco shred 1 is defined as the unshielded tobacco shred in the overlapped tobacco shred samples, and tobacco shred 2 is the occluded tobacco shred for evaluating the COT algorithm. The sum of the area of tobacco shred 1 and tobacco shred 2 is the arbitrary actual total area of the overlapped tobacco shred. The actual detection (AD) area is the complete pixel area of the overlapped tobacco shred image calculated by the OpenCV algorithm without considering the occlusion of any overlapped tobacco shred sample. The COT detection (CD) overlapped area is the pixel area of the overlapped area calculated with the COT algorithm upon obtaining the overlapped region outline of tobacco shred 2 in the overlapped tobacco shred image.

## 4 Results

### 4.1 Implementation details

#### 4.1.1 Experimental platform

The experiment in this paper was performed on a Windows 10 operating system. The GPU used was the GeForce GTX 3080 (10GB video memory), the processor used was the Intel Core i7-12700K CPU@3.61GHz, and the running memory was 64 GB. The model’s construction, training, and testing were implemented in Python using the PyTorch deep learning framework. In addition, the CUDA 11.0 parallel computing framework was used alongside the Pycharm development environment.

#### 4.1.2 Details of segmentation model training

For the overlapped tobacco shred dataset, the training set images were randomly shuffled before input to reduce the influence of the image sequence on the model. During the model training process, the batch size for model training was set to 8, the number of training arguments was taken as 60, and the initial learning rate was taken as 0.08. In the model gradient optimization, the gradient descent was performed after multiple iterations using the stochastic gradient descent (SGD) optimizer and the learning rate was attenuated during the model training process to obtain better segmentation performance.

### 4.2 Results of the segmentation model

#### 4.2.1 Improved Mask RCNN performance test

**Figure 10:**
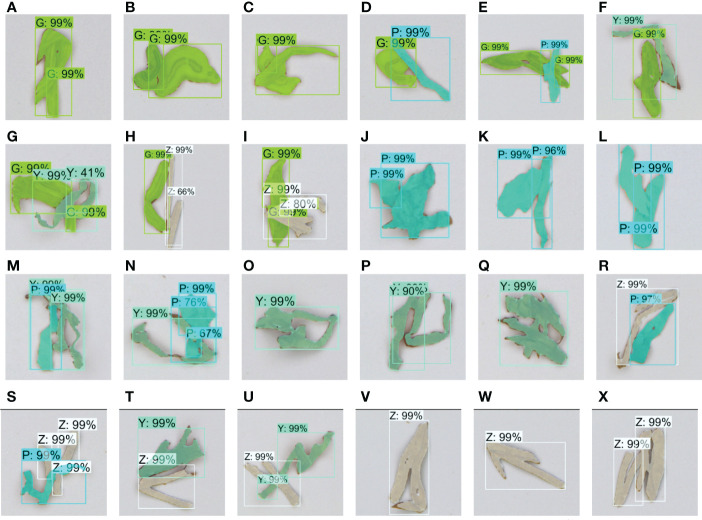
**A–X** are recognition results of 24 overlapped tobacco shreds, including self-winding, adhesion, and inter-overlapped GG, PP, YY, and ZZ, and adhesion and inter-overlapped GP, GY, GZ, PY, ZP, and ZY. G, cut stem; P, expanded tobacco silk; Y, tobacco silk; Z, reconstituted tobacco shred; GG, cut stem and cut stem; GP, cut stem and expanded tobacco silk; GY, cut stem and tobacco silk; GZ, cut stem and reconstituted tobacco shred; PP, expanded tobacco silk and expanded tobacco silk; PY, expanded tobacco silk and tobacco silk; PZ, expanded tobacco silk and reconstituted tobacco shred; YY, tobacco silk and tobacco silk; YZ, tobacco silk and reconstituted tobacco shred; ZZ, reconstituted tobacco shred and reconstituted tobacco shred.

The overlapped tobacco shred datasets were used for network training and testing using the improved Mask RCNN. **Figure 10** shows the segmentation effect diagrams for 24 overlapped types of tobacco shreds. The proposed method can accurately classify self-winding and adhesion tobacco shreds. The average target recognition accuracy for the four self-winding tobacco shreds was 99% (A99%, J99%, O99%, and V99%). The average object recognition accuracy for overlapped tobacco shreds across nine adhesion types was 94% (B99%, D99%, F99%, H66%, K96%, M99%, P90%, T99%, and W99%). The average target recognition accuracy for the inter-overlapped tobacco shreds that were difficult to segment was 86.9% (C99%, E99%, G41%, I80%, L99%, N67%, Q99%, S99%, and X99%). Therefore, the above analysis indicates that various overlapped tobacco shreds can be accurately identified using the improved Mask RCNN. Although the improved network leads to increased training and inference time, it can still ensure accurate segmentation and recognition relatively quickly.

The experimental results show that after 60 training rounds, the loss and mAP of the model remained stable, the training time was 4454.8 s, and the inference time was 0.04 s. The average precision (*AP* ^50^) and average recall (*AR^s^*) for the object detection performance of the improved Mask RCNN model were 90.2% and 75.2%, respectively. Additionally, the *AP* ^50^ and *AR^s^* for the segmentation performance of the Mask RCNN model were 89.1% and 73.2%, respectively. The improved network performance is shown in **Table 2**. The segmentation model performance is separately explained below.

**Table 2:** Improved Mask region-based convolutional neural network (RCNN) performance.

Network structure		*AP*	*AP^50^*	*AP* ^75^	*AP^s^*	*AR* ^10^	*AR* ^s^	T-time (s)	P-time (s)
**Improved Mask RCNN**	**Detection**	**0.677**	**0.902**	**0.787**	**0.749**	**0.752**	**0.752**	**4454.8**	**0.044**
	**Segmentation**	**0.641**	**0.891**	**0.733**	**0.728**	**0.732**	**0.732**		

Mask RCNN, mask region-based conventional neural network.

#### 4.2.2 Performance test of the Densenet121

This section evaluates the model performance of the DenseNet121 for segmenting overlapped tobacco shreds. The following networks were selected as baseline backbones: Vgg11 (Simonyan and Zisserman, 2014), MobileNet (Howard et al., 2017), Resnet50 (He et al., 2016), Resnet101 (Xu et al., 2020), and Densenet121 (Huang et al., 2017). The performance index comparison of different CNN backbone networks is shown in **Table 3**.

**Table 3:** The performance index comparison of different convolutional neural network (CNN) backbone networks.

Network Structure	*AP*	*AP^50^*	*AP* ^75^	*AP^s^*	*AR* ^10^	*AR* ^s^	T-time (s)	P-time (s)
Mask RCNN Vgg11	0.523	0.817	0.547	0.646	0.648	0.648	4610.0	0.053
Mask RCNN MobileNet	0.602	0.840	0.661	0.695	0.697	0.697	3986.2	0.016
Mask RCNN ResNet 50	0.466	0.805	0.476	0.582	0.583	0.583	3725.4	0.035
Mask RCNN ResNet 101	0.533	0.834	0.590	0.640	0.642	0.642	4771.3	0.043
**Mask RCNN Densenet 121**	**0.612**	**0.861**	**0.666**	**0.698**	**0.695**	**0.695**	**4466.9**	**0.041**

Mask RCNN, mask region-based conventional neural network.

**Table 3** compares the Mask RCNN using Densenet121 as the backbone with Mask RCNN using lightweight networks such as ResNet50 and MobileNet as the backbone. Although the training time (4466.9s) and inference time (0.041s) of the DenseNet121 were longer than those of the ResNet50 (3725.4s and 0.035s, respectively) and the MobileNet (3986.2s and 0.016s, respectively), DenseNet121 had the best *AP* ^50^ and *AR^s^* at 0.861 and 0.695, respectively, and the remaining indices were also the better than or comparable to the other backbones. The training time and inference time of the DenseNet121 backbone network were minimal, and the performance indicators were the best when compared with the Mask RCNN using Vgg11 or ResNet101 as the backbone. This means that in lightweight and deep networks, Mask RCNN with the DenseNet121 backbone network can effectively identify and segment overlapped tobacco shreds.

#### 4.2.3 Performance test based on U-FPN

Based on the improved proof in 3.2.2, the model performance for small-target detection based on the U-FPN algorithm was evaluated. The performance indices of Mask RCNN–FPN, Mask RCNN–U-FPN, and Mask RCNN–U-FPN–Densenet121 are listed in **Table 4**.

**Table 4:** The performance index comparison of different feature pyramid networks (FPNs).

Network structure	*AP*	*AP^50^*	*AP* ^75^	*AP^s^*	*AR* ^10^	*AR* ^s^	T-time (s)	P-time (s)
Mask RCNN–FPN	0.466	0.805	0.476	0.582	0.583	0.583	3725.4	0.035
Mask RCNN–U-FPN	0.498	0.819	0.493	0.609	0.608	0.608	0.608	0.608
**Mask RCNN–U-FPN–Densenet121**	**0.637**	**0.877**	**0.689**	**0.724**	**0.727**	**0.727**	**4523.1**	**0.042**

Mask RCNN, mask region-based conventional neural network.

**Table 4** indicates that the Mask RCNN–U-FPN network shows noticeable improvements in small-target detection and segmentation performance compared with the Mask RCNN-FPN network, which proves that even when the backbone was the ResNet50 network, U-FPN showed excellent network performance. Compared with the Mask RCNN–U-FPN network, Mask RCNN–U-FPN–Densenet121 demonstrated effective network performance improvements without significantly increasing the network training and prediction time. The *AP* ^50^ and *AR^s^* of the Mask RCNN–U-FPN–Densenet121 network were the best recorded, at 0.877 and 0.727, respectively. Except for time, the other indicators were still the best in the Mask RCNN–U-FPN–Densenet121 network, which proves U-FPN’s effectiveness.

#### 4.2.4 Performance test based on RPN

**Figure 11:**
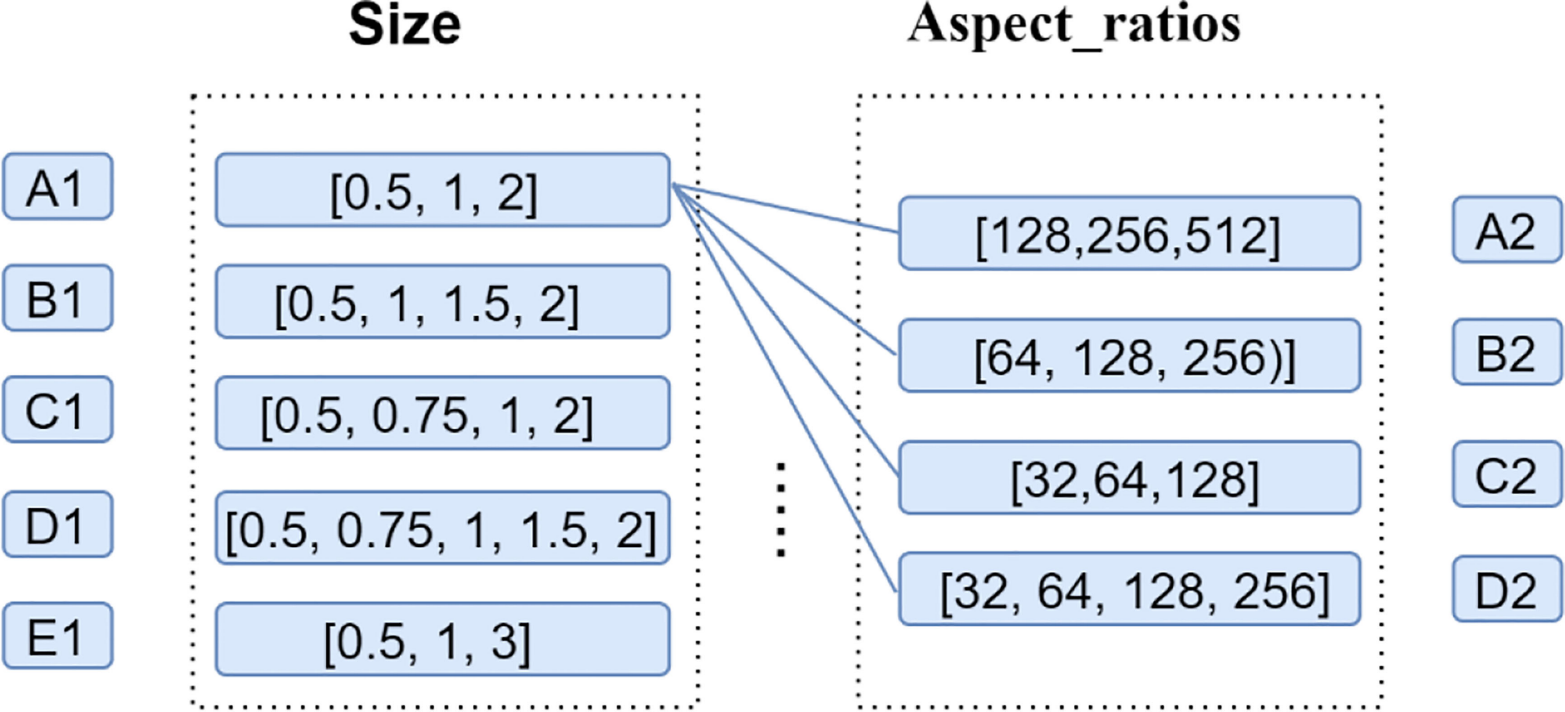
A series of Anchor sizes (A1–E1) and aspect ratios (A2–E2).

In this section, Mask RCNN–U-FPN–Densenet121 is referred to as P-Mask RCNN for convenience. A series of Anchor sizes (A1–E1) and aspect ratios (A2–E2) was designed on the premise that the aspect ratios of all images was [0, 0.5, 0.63, 0.8, 1.0, 1.26, 1.59, 2.0], as shown in **Figure 11**. Additionally, the anchors parameters in the RPN of the P-Mask RCNN model were adjusted, the overlapped tobacco shred datasets were inputted, and the model’s network performance was compared. **Table 5** shows different P-Mask RCNN performances under different sizes and aspect ratios. In **Table 5**, the P-Mask RCNN-A1A2 represents the original network with the default anchors parameters. The networks with adjusted anchors parameters range from P-Mask RCNN-A1B2 to P-Mask RCNN-E1D2.

**Table 5:** The performance index comparison of different sizes and aspect ratios.

Network parameter	*AP*	*AP^50^*	*AP* ^75^	*AP^s^*	*AR* ^10^	*AR* ^s^	T-time (s)	P-time(s)
P-**Mask RCNN-A1A2**	**0.636**	**0.877**	**0.689**	**0.724**	**0.727**	**0.727**	**4523.1**	**0.042**
P-Mask RCNN-A1B2	0.628	0.884	0.717	0.713	0.715	0.715	4528.2	0.041
P-Mask RCNN-A1C2	0.612	0.874	0.673	0.699	0.701	0.702	4473.1	0.043
P-Mask RCNN-A1D2	0.632	0.888	0.702	0.709	0.712	0.712	4436.0	0.04
P-**Mask RCNN-B1A2**	**0.605**	**0.862**	**0.650**	**0.688**	**0.693**	**0.693**	**4441.5**	**0.042**
P-**Mask RCNN-B1B2**	**0.647**	**0.862**	**0.710**	**0.721**	**0.724**	**0.724**	**4466.2**	**0.041**
P-**Mask RCNN-B1C2**	**0.614**	**0.860**	**0.666**	**0.716**	**0.718**	**0.718**	**4630.5**	**0.041**
P-**Mask RCNN-B1D2**	**0.641**	**0.891**	**0.733**	**0.728**	**0.732**	**0.732**	**4454.8**	**0.044**
P-Mask RCNN-C1A2	0.615	0.877	0.648	0.709	0.711	0.711	4438.8	0.042
P-Mask RCNN-C1B2	0.632	0.875	0.708	0.718	0.720	0.720	4448.6	0.041
P-Mask RCNN-C1C2	0.627	0.877	0.699	0.712	0.715	0.715	4432.5	0.042
P-Mask RCNN-C1D2	0.635	0.871	0.711	0.717	0.719	0.719	5220.2	0.041
P-Mask RCNN-D1A2	0.641	0.873	0.736	0.732	0.735	0.735	4430.3	0.041
P-Mask RCNN-D1B2	0.642	0.871	0.735	0.728	0.730	0.730	5127.5	0.041
P-Mask RCNN-D1C2	0.633	0.873	0.697	0.720	0.723	0.723	4593.7	0.042
P-Mask RCNN-D1D2	0.634	0.882	0.706	0.720	0.723	0.723	4568.4	0.041
P-Mask RCNN-E1A2	0.628	0.876	0.699	0.714	0.716	0.716	4735.7	0.042
P-**Mask RCNN-E1B2**	**0.625**	**0.867**	**0.701**	**0.713**	**0.716**	**0.715**	**4556.7**	**0.043**
P-Mask RCNN-E1C2	0.634	0.871	0.735	0.718	0.720	0.720	4481.3	**0.04**
P-**Mask RCNN-E1D2**	**0.610**	**0.861**	**0.670**	**0.693**	**0.696**	**0.696**	**4447.3**	**0.041**

Mask RCNN, mask region-based conventional neural network.

**Table 5** shows that choosing inappropriate anchors parameters, such as B1A2, B1B2, B1C2, E1B2, and E1D2, led to a decline in network performance, and this proves the importance of improving parameters. In addition, it indicates that changing the anchors parameter can effectively improve the network performance at the cost of a slight increase in training time. Finally, it was determined that the Anchor sizes [0.5, 1, 1.5, 2] and the aspect ratios [32, 64, 128, 256] performed the best. Although the training time and prediction time of the P-Mask RCNN-B1D2 network were slightly higher than with other anchors parameters, performance indices were better.

#### 4.2.5 Comparison with other instance segmentation methods

**Figure 12:**
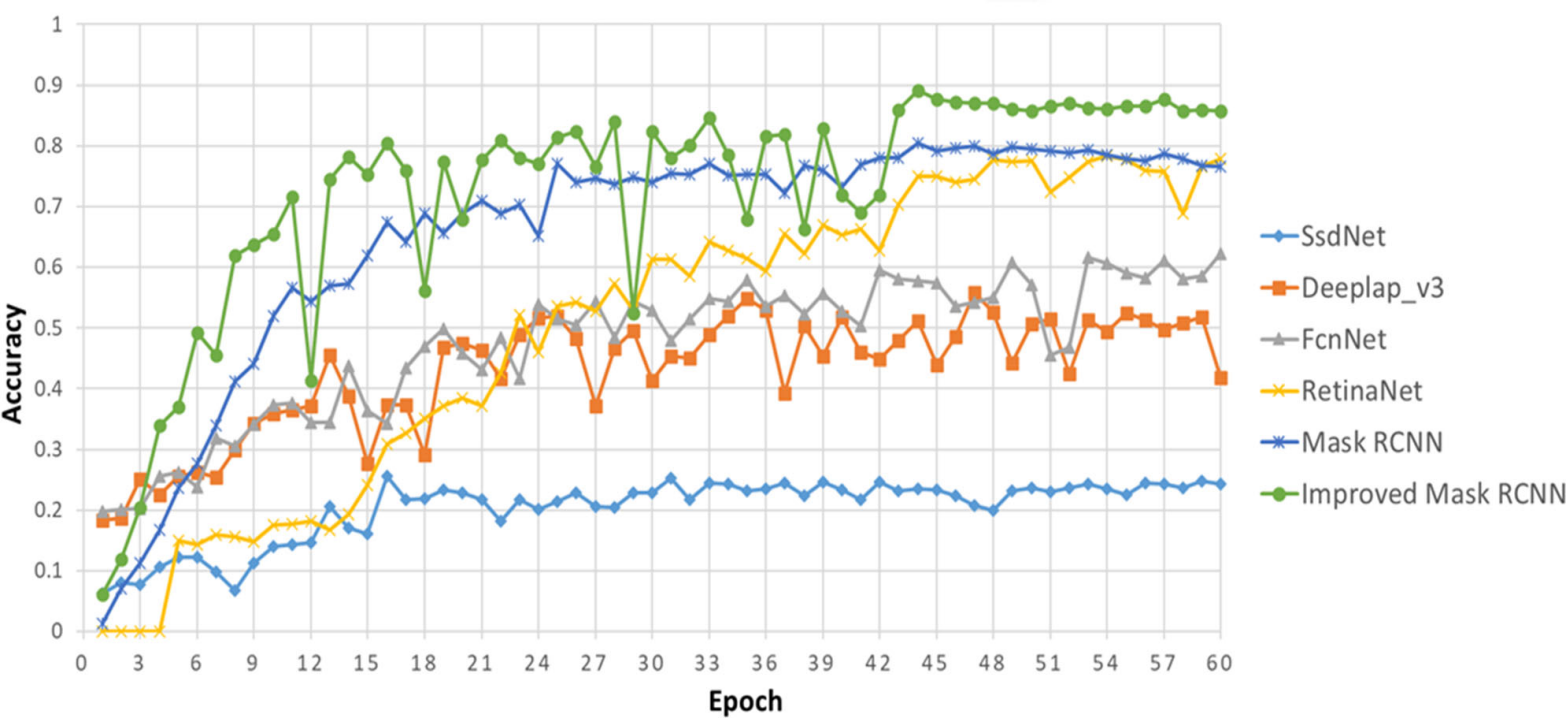
Multi-network performance comparison about *AP* ^50^.

In order to further prove the effectiveness of the improved Mask RCNN at solving the problem of overlapped tobacco shred image segmentation, SsdNet (Liu et al., 2016), Deeplap_v3 (He et al., 2022), FcnNet (Wu et al., 2022), RetinaNet (Lin et al., 2017), and Mask RCNN were selected as baseline models. The performance index obtained is shown in **Table 6** and **Figure 12**.

**Table 6:** The performance index comparison of different instance segmentation methods.

Network structure	*AP*	*AP* *^50^*	*AP* ^75^	*AP* *^s^*	*AR* ^10^	*AR* ^s^	T-time (s)	P-time (s)
SsdNet	0.111	0.246	0.082	0.111	0.337	0.362	1187.2	0.01
Deeplap_v3	0.368	0.558	0.312	0.351	0.491	0.491	2616.4	0.024
FcnNet	0.391	0.623	0.347	0.383	0.532	0.532	2047.0	0.026
RetinaNet	0.416	0.783	0.370	0.416	0.575	0.589	2484.4	0.033
Mask RCNN	0.466	0.805	0.476	0.582	0.583	0.583	3725.4	0.035
**Improved Mask RCNN**	**0.641**	**0.891**	**0.733**	**0.728**	**0.732**	**0.732**	**4454.8**	**0.044**

Mask RCNN, mask region-based conventional neural network.

**Table 6** and **Figure 12** show that compared with the SsdNet, Deeplap_v3, FcnNet, and RetinaNet models, Mask RCNN demonstrated the best performance, with an *AP* ^50^ and *AR^s^* of approximately 0.805 and 0.583, respectively. Other indicators were also better in Mask RCNN, which proves that Mask RCNN can best solve the problem of overlapped image segmentation. Compared with the Mask RCNN network, the improved Mask RCNN network achieved a better performance, with an *AP* ^50^ and *AR^s^* of 0.891 and 0.732, respectively, which represents an improvement of 8.6% and 14.9%, respectively, although there was also an increase in training time and inference time. In summary, the improved Mask RCNN proposed in this study performed the best of the baseline networks studied and can effectively and accurately carry out image segmentation of overlapped tobacco shreds.

### 4.3 Evaluation of the COT algorithm

This paper proposes an algorithm for the overlapped region and calculating the overlapped area to obtain an accurate estimation of the overlapped area in the covered tobacco shred, hence overcoming the issues of obtaining the overlapped region in the covered tobacco shred and the failure to detect the overlapped area when different types of tobacco shred overlap. Twenty samples from four tobacco shred types were selected to develop the original tobacco shred sample set, as shown in **Table 7**. It can be seen that Y-1 to Y-5 are five tobacco silk samples with different shapes, G-1 to G-5 are five different shapes of cut stem samples, P-1 to P-5 are five samples of expanded tobacco silk with different shapes, and Z-1 to Z-5 are five samples of reconstituted tobacco shred with varying shapes. The actual tobacco shred area in each of the 20 samples obtained by the OpenCV algorithm is shown in **Table 8**.

**Table 7:** The original tobacco shred sample datasets.

Type of sample	1	2	3	4	5
Y	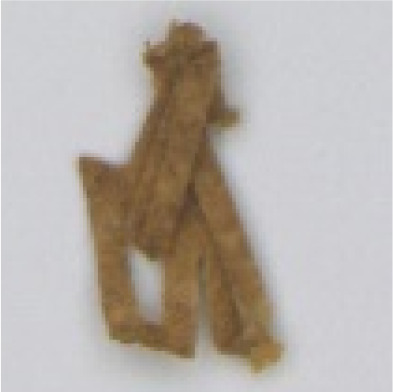	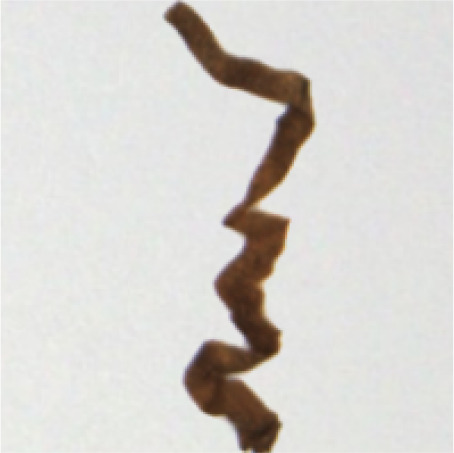	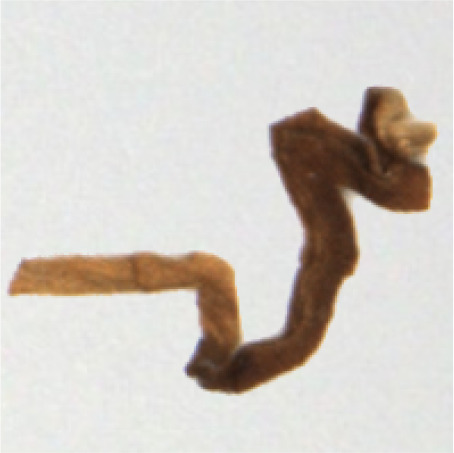	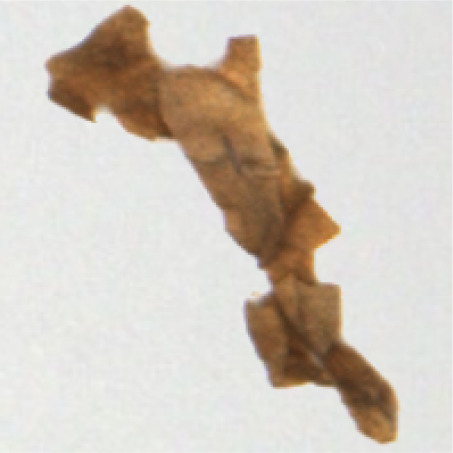	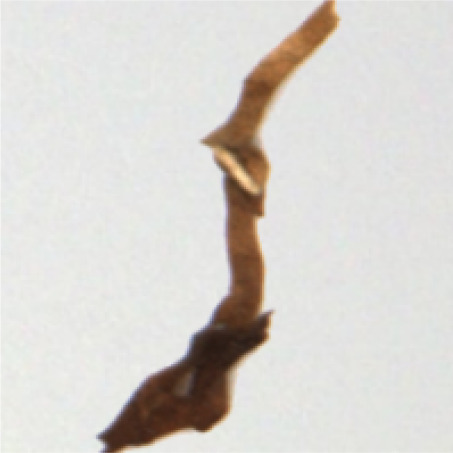
G	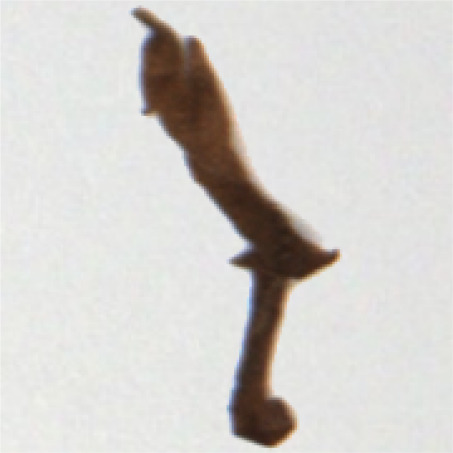	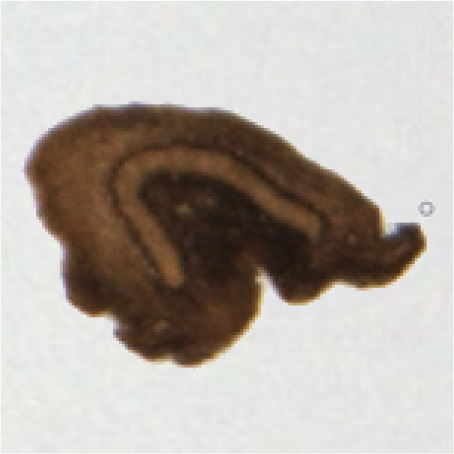	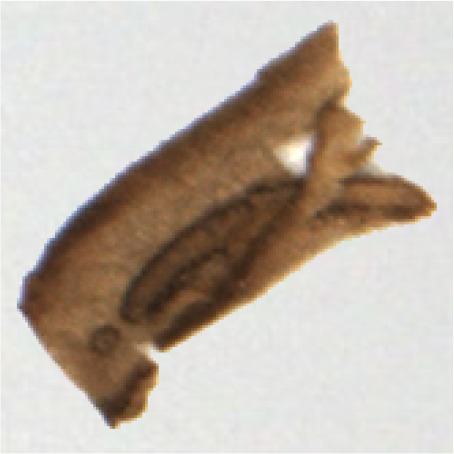	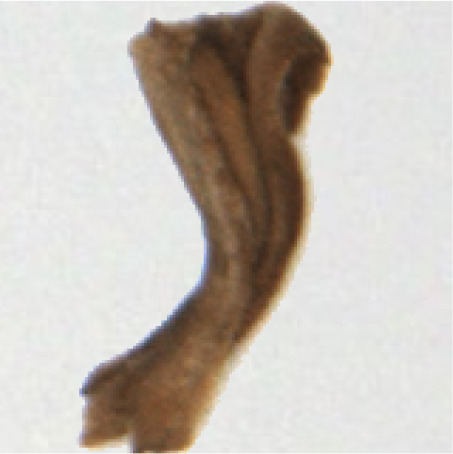	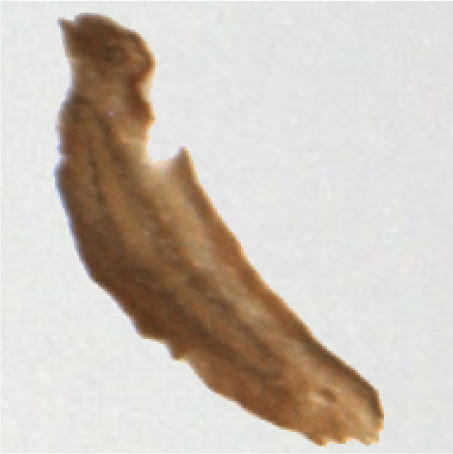
P	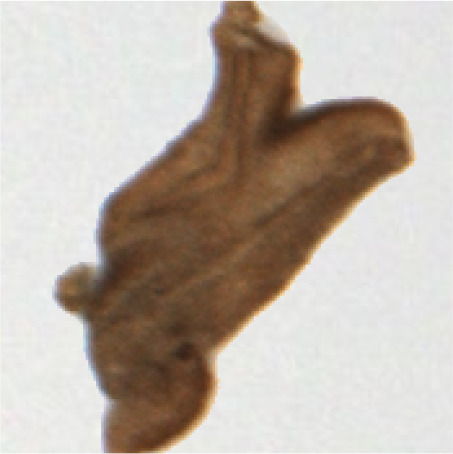	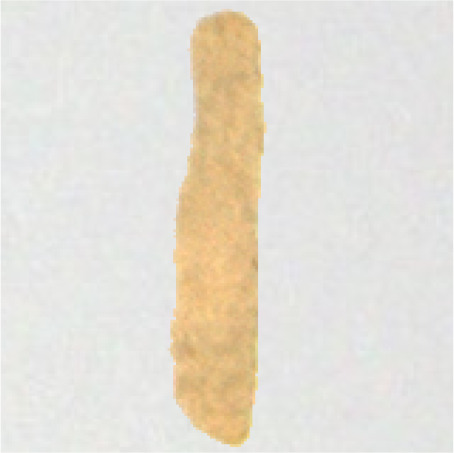	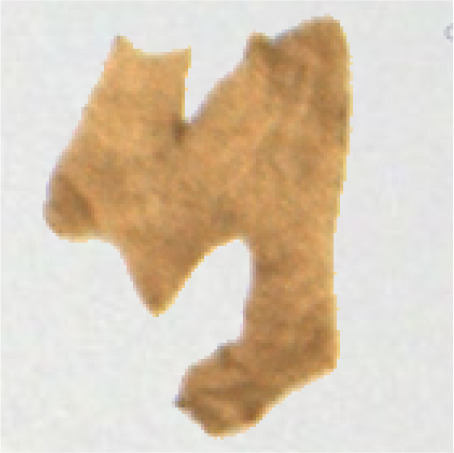	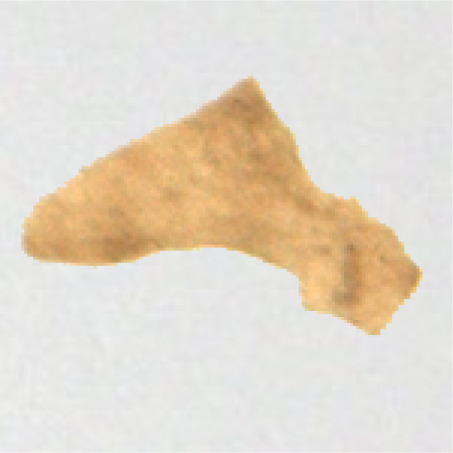	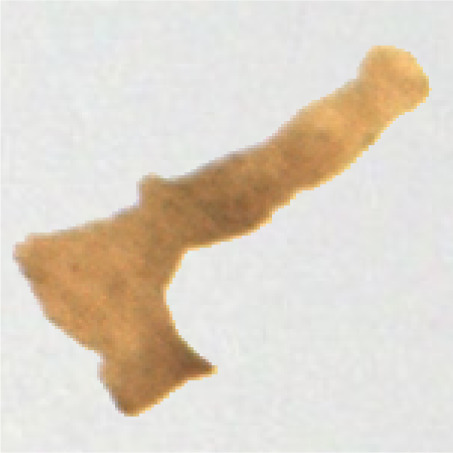
Z	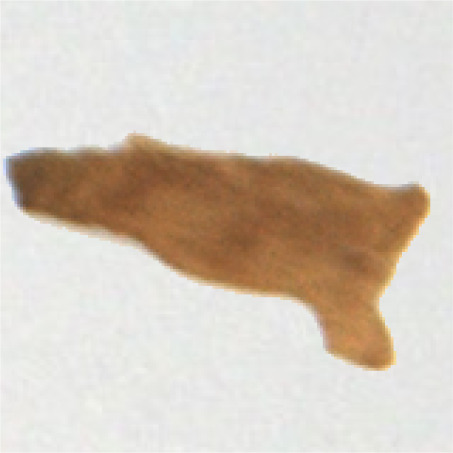	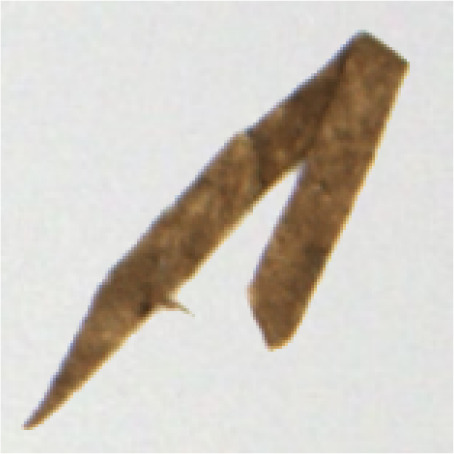	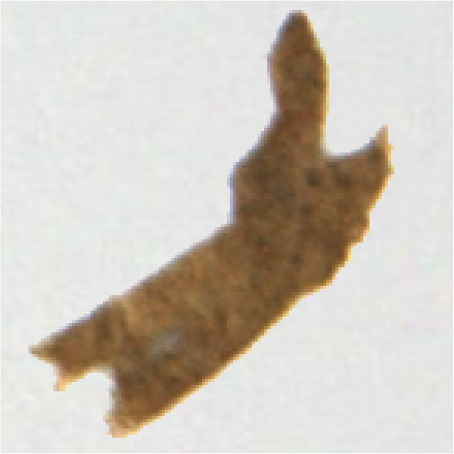	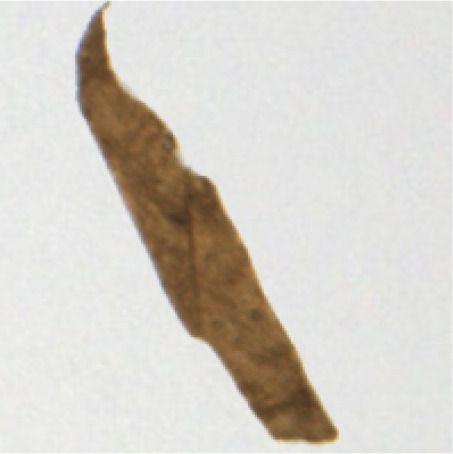	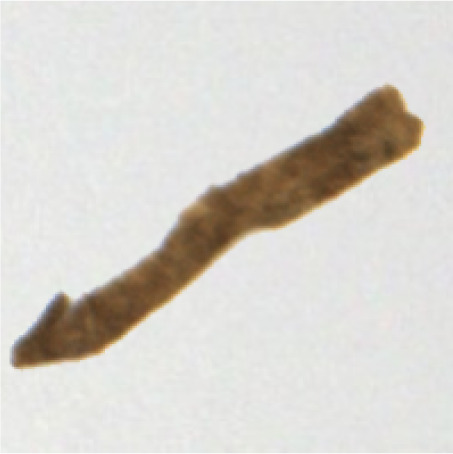

G, cut stem; P, expanded tobacco silk; Y, tobacco silk; Z, reconstituted tobacco shred.

**Table 8:** The actual area of tobacco shred in each of the 20 samples.

Area (category)	1	2	3	4	5
Y	6611	8952	9747.5	8243	5956
G	7650	9692.5	7359.5	9813	11227
P	3917.5	7227.5	4999	4861	6277.5
Z	7305	5289.5	8593.5	5464.5	6706.5

G, cut stem; P, expanded tobacco silk; Y, tobacco silk; Z, reconstituted tobacco shred.

In this study, 24 various overlapped samples based on the 20 original tobacco shred sample sets were constructed, as shown in **Table 9**. The abscissa is the serial number (1–4) of the tobacco shred sample, and the vertical coordinate is the type of overlapped tobacco shred (GY, GZ, PG, PY, PZ, and YZ).

**Table 9:** The 24 overlapped tobacco shred samples with different overlapped types.

Type of overlapped sample	1	2	3	4
GY	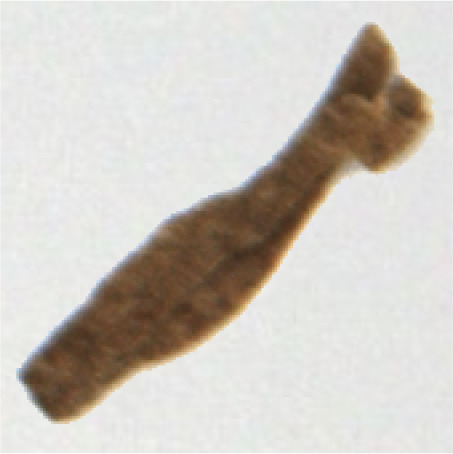	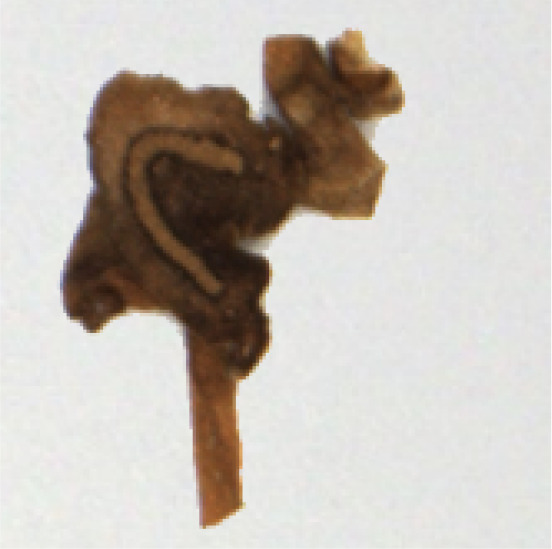	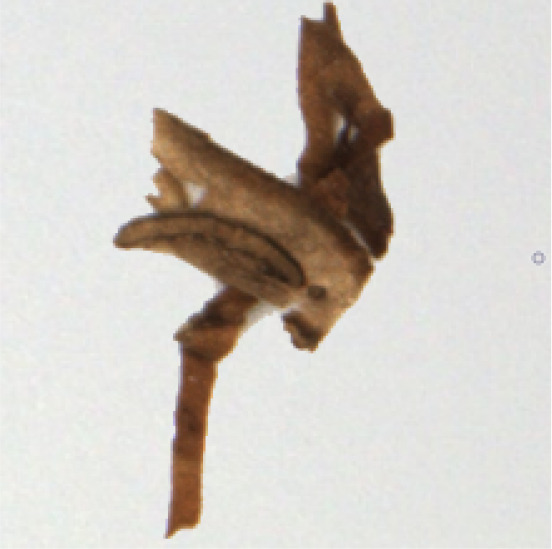	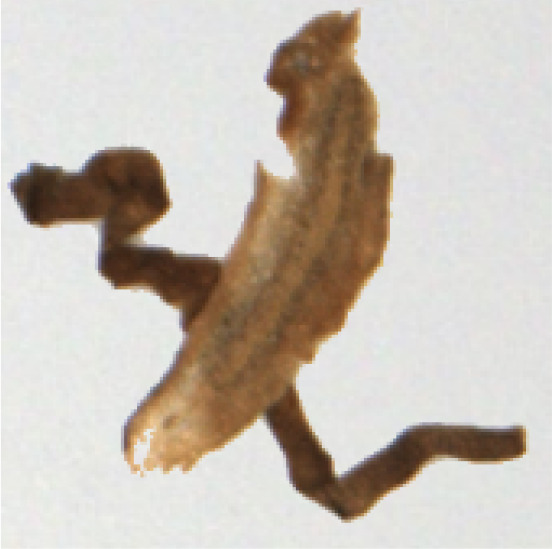
GZ	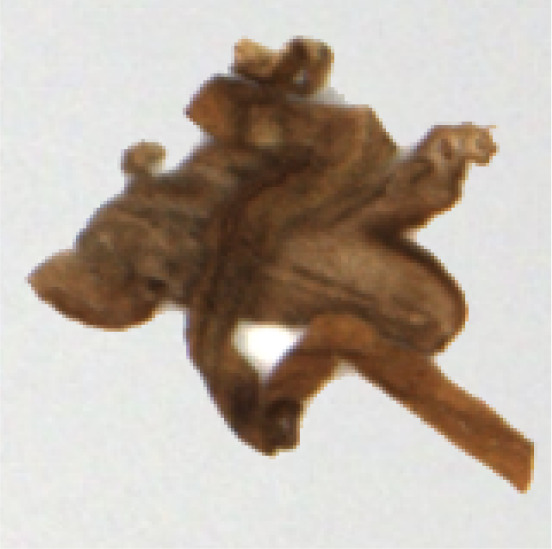	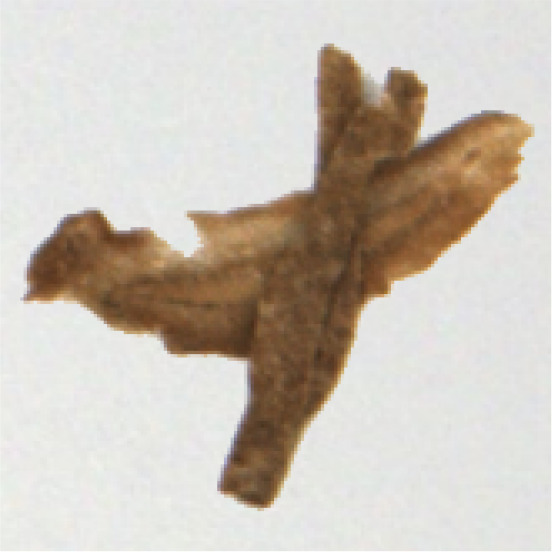	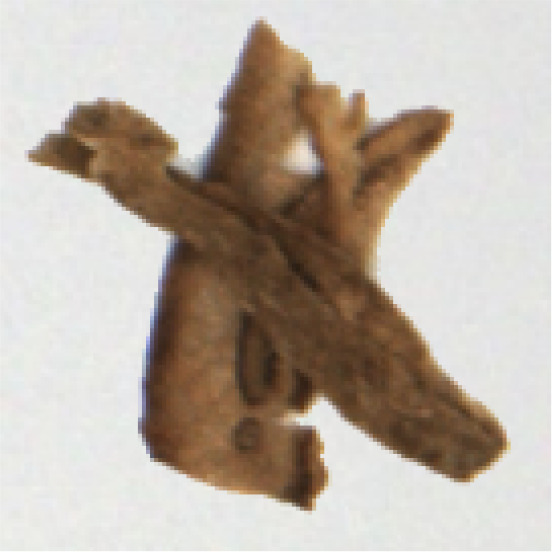	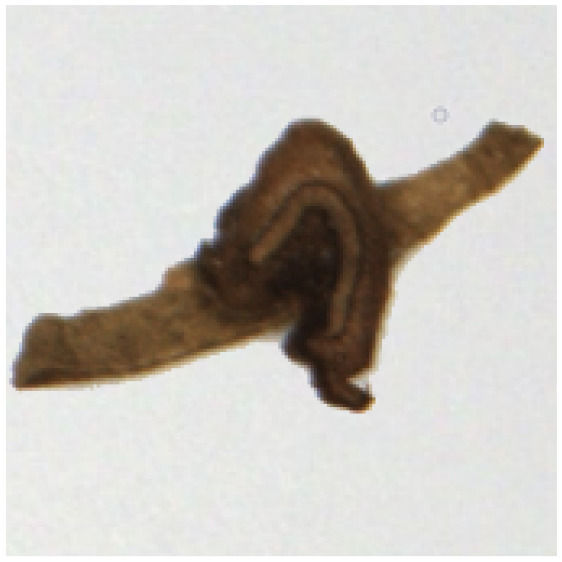
PG	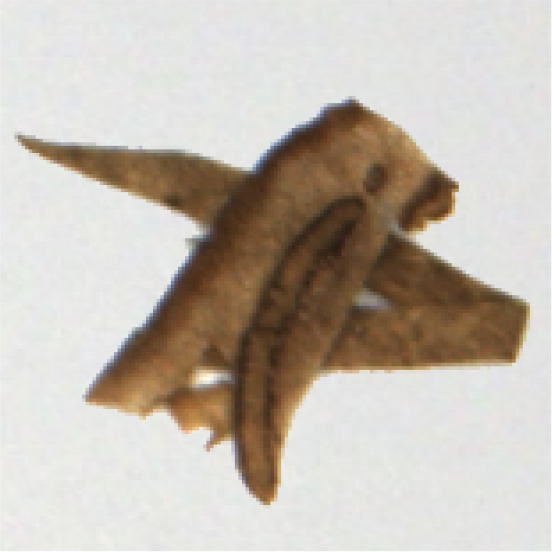	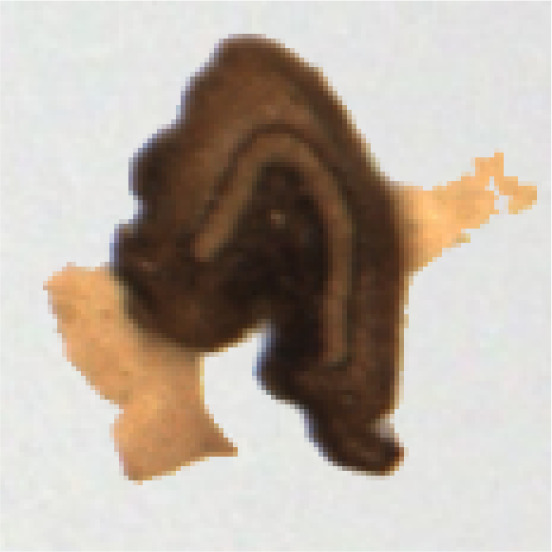	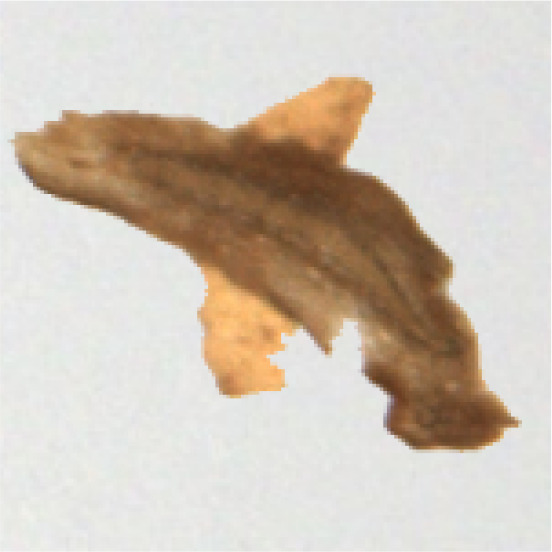	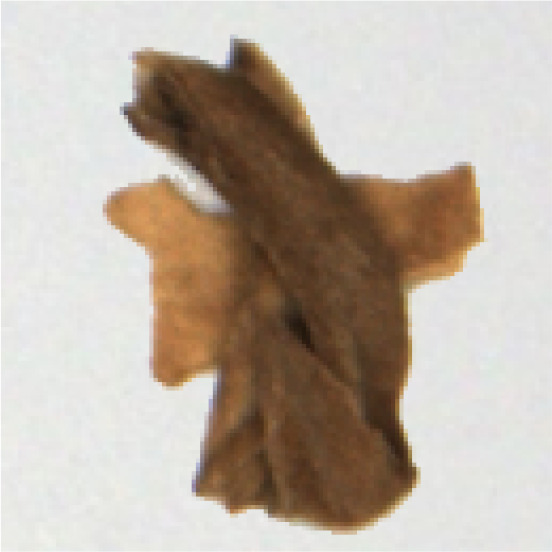
PY	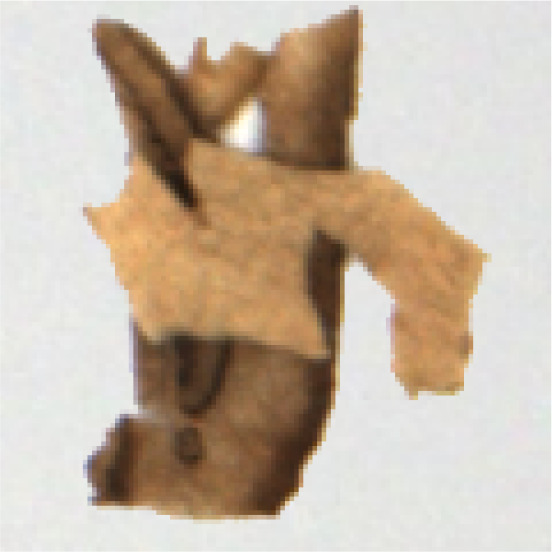	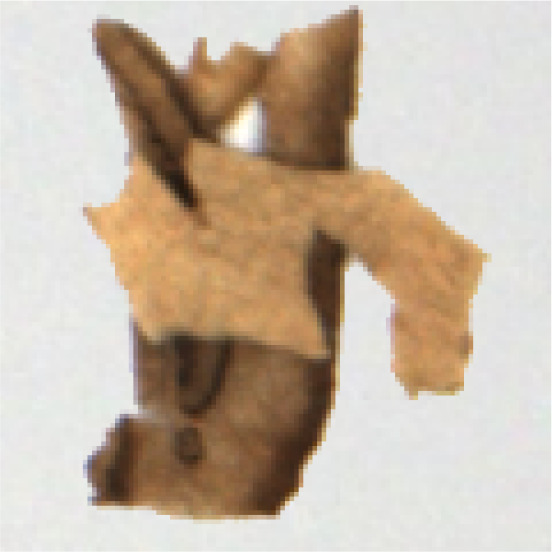	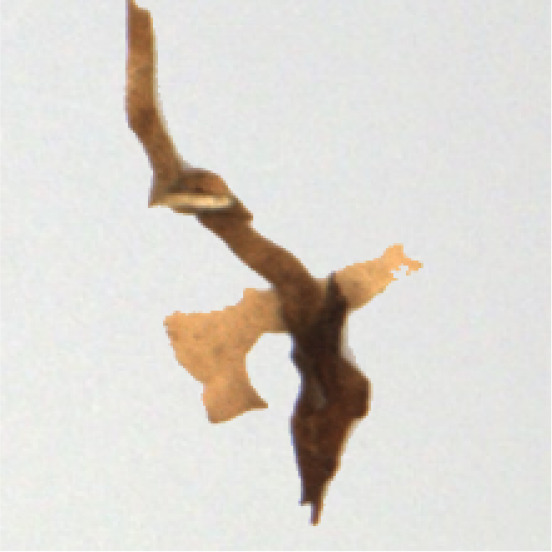	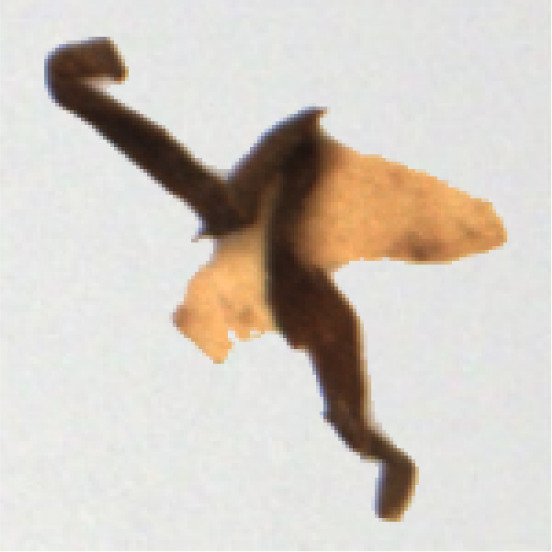
PZ	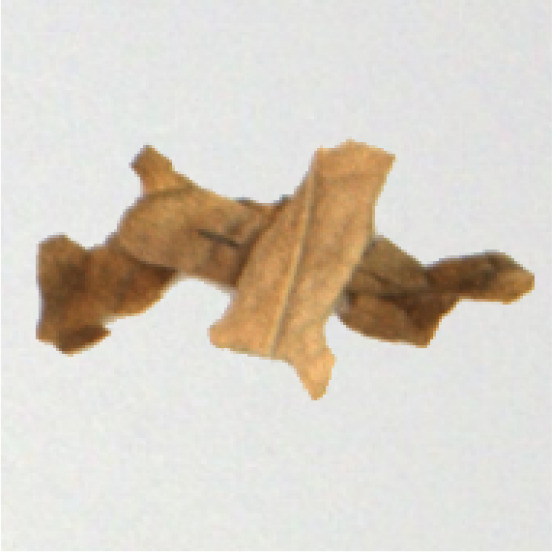	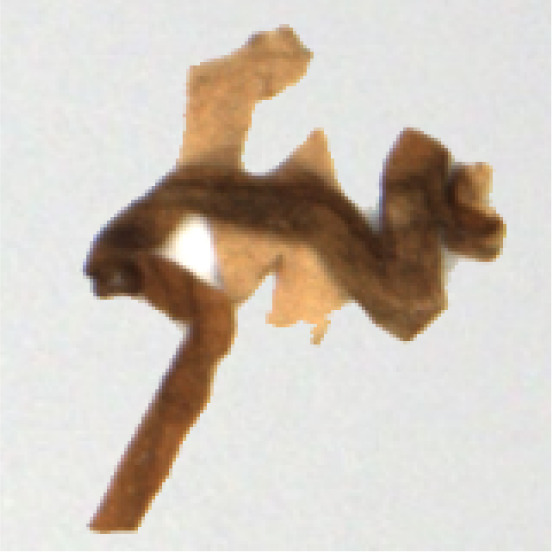	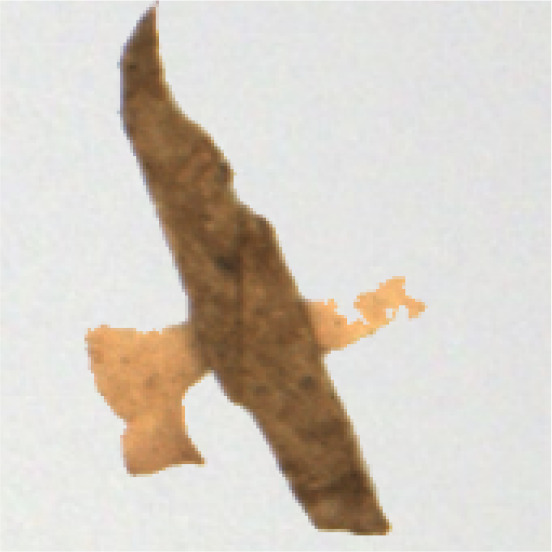	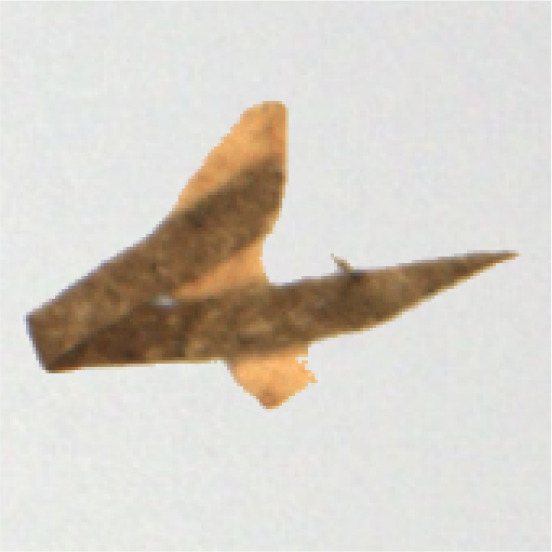
YZ	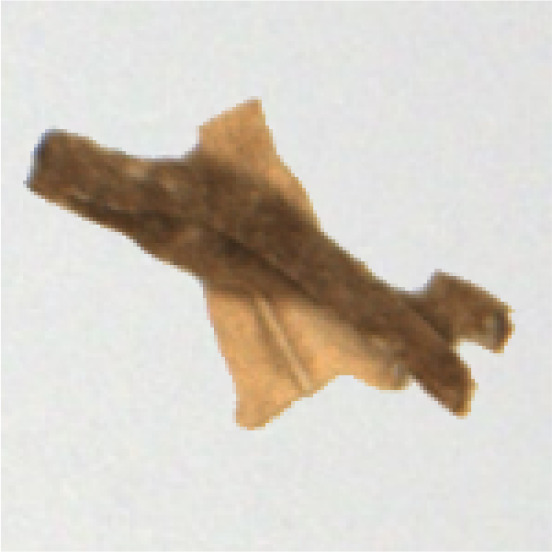	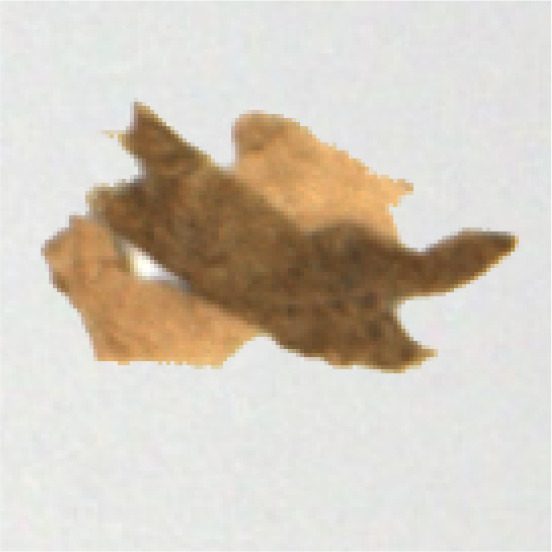	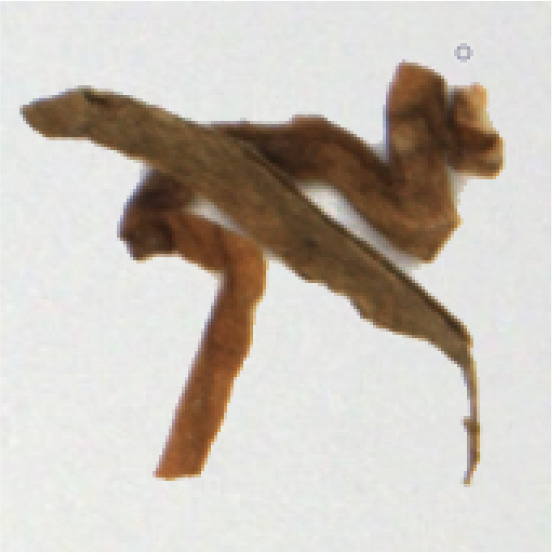	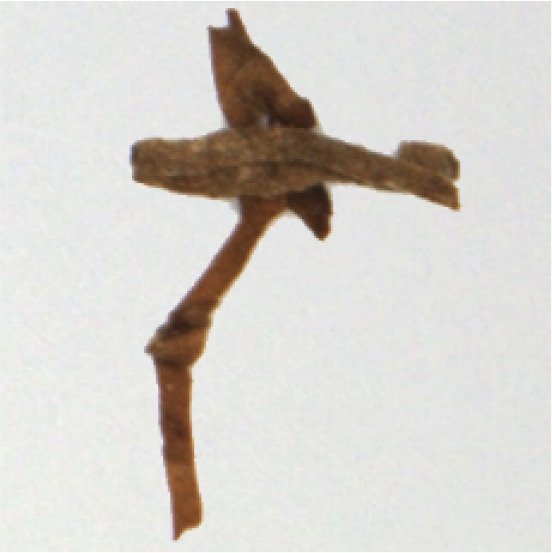

GP, cut stem and expanded tobacco silk; GY, cut stem and tobacco silk; GZ, cut stem and reconstituted tobacco shred; PY, expanded tobacco silk and tobacco silk; PZ, expanded tobacco silk and reconstituted tobacco shred; YZ, tobacco silk and reconstituted tobacco shred.

The outline of the overlapped region in the covered tobacco shred was obtained, and its area was calculated using the COT algorithm for 24 different random tobacco shred overlap types. The experimental results are shown in **Table 10**.

**Table 10:** Pixel area of the 24 overlapped tobacco shred samples under different random overlapping conditions.

Serial number	Sample	Tobacco shred 1	Tobacco shred 2	Area_1 + Area_2	AAR	CAR
**1**	**GY-1**	**G-1**	**Y-2**	**16602.0**	**0.771**	**0.789**
**2**	**GY-2**	**G-2**	**Y-4**	**17935.5**	**0.933**	**1.05**
**3**	**GY-3**	**G-4**	**Y-1**	**16424**	**0.922**	**1**
4	GY-4	G-5	Y-2	20179	0.842	0.898
5	GZ-1	G-4	Z-5	16519.5	0.846	0.952
6	GZ-2	G-2	Z-5	16399	0.8	0.884
**7**	**GZ-3**	**G-1**	**Z-4**	**13114.5**	**0.842**	**1**
8	GZ-4	G-2	Z-1	16997.5	0.795	0.901
9	PG-1	P-4	G-1	12511	0.758	0.857
10	PG-2	P-3	G-4	14812	0.844	0.956
11	PG-3	P-5	G-3	13637	0.844	0.935
**12**	**PG-4**	**P-2**	**G-2**	**16920**	**0.684**	**0.81**
13	PY-1	P-4	Y-4	13104	0.849	0.916
14	PY-2	P-3	Y-1	11610	0.748	0.799
15	PY-3	P-5	Y-3	16025	0.825	0.947
16	PY-4	P-2	Y-2	16179.5	0.757	0.804
17	PZ-1	P-4	Z-3	13454.5	0.886	0.983
18	PZ-2	P-3	Z-1	12304	0.772	0.852
19	PZ-3	P-5	Z-5	12984	0.719	0.814
20	PZ-4	P-2	Z-2	12517	0.715	0.806
21	YZ-1	Y-2	Z-3	17545.5	0.742	0.802
22	YZ-2	Y-4	Z-5	14949.5	0.871	0.964
23	YZ-3	Y-1	Z-5	13317.5	0.866	0.955
24	YZ-4	Y-2	Z-3	17545.5	0.867	0.917
**Average**					**0.812**	**0.9**

AAR, actual area ratio; CAR, COT area ratio. G, cut stem; P, expanded tobacco silk; Y, tobacco silk; Z, reconstituted tobacco shred; GG, cut stem and cut stem; GP, cut stem and expanded tobacco silk; GY, cut stem and tobacco silk; GZ, cut stem and reconstituted tobacco shredPY, expanded tobacco silk and tobacco silk; PZ, expanded tobacco silk and reconstituted tobacco shred; YZ, tobacco silk and reconstituted tobacco shred.

**Table 10** indicates that among the total area of overlapped tobacco shreds calculated by the OpenCV algorithm, the PG-4 overlapped type sample showed the worst area detection effect, with an AAR of 0.648, whereas the GY-2 overlapped type sample showed the best area detection effect, with an AAR of 0.933. The average actual area ratio detected of overlapped tobacco shred areas was 0.812. However, it can be seen that there is still a large discrepancy between the observed overlapped total area in the tobacco shreds, and the actual area, because there is a missing overlapped area in the covered tobacco shred. The overlapped area obtained by the COT algorithm effectively makes up for the lack of the total area. Moreover, the GY-1 overlapped type had the worst area detection effect, with a CAR of 0.789. The best area detection effect, with a detection rate of 1%, was shown for the GY-3 and GZ-3 overlapped types. The average area detection rate of the overlapped tobacco shred reached 0.90. The worst and best area detection increase rates of CAR compared with AAR were 1.8% and 15.8%, respectively. The average area detection increase rate was 8.8%. In addition, the COT algorithm was applied to 24 experimental sets, and no negative optimization occurred, showing that the algorithm had excellent performance and was effective.

## 5 Conclusion

This study develops an improved Mask RCNN segmentation model with a COT algorithm to overcome the problems of having many types of overlapped tobacco shreds, difficulty in the segmentation of small overlapped tobacco shred objects, and obtaining overlapped region and area calculation. The proposed model can be used to calculate the area of overlapped parts in tobacco shred images. Within the study context, this model was successfully applied to the instance segmentation and area calculation of overlapped tobacco shreds. Based on the aforementioned statements, the following innovations were achieved:

1. A database of 920 overlapped tobacco shred images and two original overlapped tobacco shred image datasets was developed to segment overlapped tobacco shred types, effectively avoiding overfitting and ensuring suitability for actual field use.
2. An improved Mask RCNN network was proposed by adopting DenseNet121 as the backbone, adding upsampling and connection with C2 and C3 level as U-FPN structure, and optimizing anchors parameters, effectively improving the segmentation accuracy for overlapped tobacco shred images. The DenseNet121 model improves the ability of the Mask RCNN network to extract tiny features in the shallow information of overlapped tobacco shreds. The utilization rate of the shallow information and extracted tiny features of tobacco shreds was enhanced with the U-FPN structure. anchors parameters were optimized to reduce both the failure to detect tobacco shreds and redundant calculations.
3. A COT algorithm was proposed to obtain the overlapped tobacco shred region and calculate the overlapped area, which avoids the loss in the overlapped region area. The COT algorithm significantly improves the detection accuracy of the total area of the overlapped tobacco shreds without negative optimization.

Accordingly, the method proposed in this paper can accurately perform image segmentation of overlapped tobacco shreds and area calculation of the overlapped region. However, this study has several limitations, namely that the number of samples within the datasets was insufficient, the segmentation accuracy must be further improved, and the stacked tobacco shred in abnormal tobacco shred was not studied.

Follow-up work should consider the aspects below:

(1) More abnormal tobacco shreds in the production line should be collected to expand the overlapped tobacco shred datasets under different overlapped types.
(2) The effects of different geometric features of tobacco shreds, such as their length, width, area, and aspect ratios, on the segmentation of overlapped tobacco shreds must be explored. These features and image information must be input into the segmentation network to enhance the network performance of the model.
(3) Although the content of the stacked tobacco shreds is small, it still significantly impacts the accuracy of the component determination. In future studies, the stacked tobacco shreds in the abnormally shredded tobacco should also be sent to the datasets for segmentation.
(4) The scheme proposed in this paper must be installed and applied in real-world scenarios to verify the performance of the model and algorithm.

## Data availability statement

The raw data supporting the conclusions of this article will be made available by the authors, without undue reservation.

## Author contributions

LW and KJ researched the research background, proposed the methodology, designed the experiment, and wrote and revised the manuscript. YF and XX provided experimental materials and equipment, participated in the dataset shooting and designed the model test experiment. LF, WZ, and YF participated in the overlapping tobacco type experiment and supervised the model verification. QW and QN provided experimental sites and participated in the overall scheme proposal and model modification. All authors contributed to the article and approved the submitted version.
